# Nanoscale Metal–Organic Framework with an X-ray Triggerable Prodrug for Synergistic Radiotherapy and Chemotherapy

**DOI:** 10.1021/jacs.3c04602

**Published:** 2023-08-15

**Authors:** Ziwan Xu, Wenyao Zhen, Caroline McCleary, Taokun Luo, Xiaomin Jiang, Cheng Peng, Ralph R. Weichselbaum, Wenbin Lin

**Affiliations:** † Department of Chemistry, The University of Chicago, Chicago, Illinois 60637, United States; ‡ Department of Radiation and Cellular Oncology and Ludwig Center for Metastasis Research, The University of Chicago, Chicago, Illinois 60637, United States

## Abstract

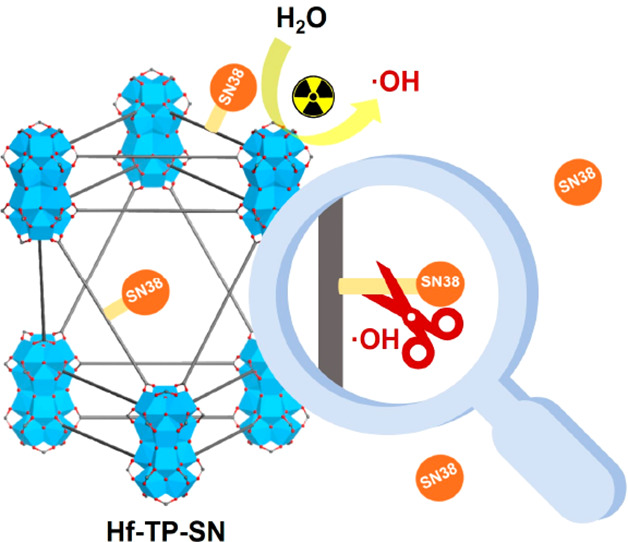

As heavy-metal-based nanoscale metal–organic frameworks (nMOFs) are excellent radiosensitizers for radiotherapy via enhanced energy deposition and reactive oxygen species (ROS) generation, we hypothesize that nMOFs with covalently conjugated and X-ray triggerable prodrugs can harness the ROS for on-demand release of chemotherapeutics for chemoradiotherapy. Herein, we report the design of a novel nMOF, Hf-TP-SN, with an X-ray-triggerable 7-ethyl-10-hydroxycamptothecin (SN38) prodrug for synergistic radiotherapy and chemotherapy. Upon X-ray irradiation, electron-dense Hf_12_ secondary building units serve as radiosensitizers to enhance hydroxyl radical generation for the triggered release of SN38 via hydroxylation of the 3,5-dimethoxylbenzyl carbonate followed by 1,4-elimination, leading to 5-fold higher release of SN38 from Hf-TP-SN than its molecular counterpart. As a result, Hf-TP-SN plus radiation induces significant cytotoxicity to cancer cells and efficiently inhibits tumor growth in colon and breast cancer mouse models.

Metal–organic frameworks (MOFs) have recently been exploited for biomedical applications due to their tunable compositions, large porosity, ease of surface functionalization, and biodegradability.^1−4^ In particular, MOFs have been widely used as drug carriers,^5−10^ via direct encapsulation of drugs in the pores,^11,12^ coordination of drugs to metal-cluster secondary building units (SBUs),^13^ and covalent conjugation of drugs to the ligands.^14^ While the first two methods can have premature drug release due to weak interactions between MOFs and drug molecules,^15^ the third method requires an actionable trigger to release the conjugated drug from the MOF.^16,17^ Among many potential triggers,^18−27^ X-ray stands out as a great external stimulus due to its deep tissue penetration,^28^ image-guided precise dosing,^29,30^ and radiotherapeutic effects through direct DNA damage or indirect cytotoxic effects via generating reactive oxygen species (ROS).^31−38^

Heavy-metal-based nanoscale MOFs (nMOFs) are excellent radioenhancers by increasing energy deposition and ROS generation.^39−41^ We hypothesized that heavy metal nMOFs with covalently conjugated drugs could be efficiently triggered by X-rays to release drugs via enhanced ROS generation and ROS-induced cleavage of drug molecules. Herein, we report the design of a Hf-TP-SN nMOF with an X-ray triggerable 7-ethyl-10-hydroxycamptothecin (SN38) prodrug for synergistic radiotherapy and chemotherapy. As a topoisomerase I inhibitor, SN38 is the active metabolite of irinotecan that has been used in the treatment of many cancers.^42,43^

We conjugated SN38 to Hf-TP-OH nMOF via the 3,5-dimethoxylbenzyl carbonate linkage, which can be cleaved by the hydroxyl radical (·OH).^38^ Hf-TP-SN was synthesized via a combination of prefunctionalization and postsynthetic modification (Figure 1). First, the dicarboxylic acid H_2_TP-OH was synthesized in five steps starting from 3,5-dimethoxylbenzyl alcohol via protection with a *tert*-butyldimethylsilyl group, lithiation, and carboxyl group installation, coupling with 2,5-dibromobenzylamine via an amide bond, Suzuki coupling with (4-(methoxycarbonyl)phenyl)boronic acid, and base-catalyzed hydrolysis (Scheme S1). Next, Hf-TP-OH with Hf_12_ SBUs was synthesized via a solvothermal reaction between HfCl_4_, H_2_TP-OH, trifluoracetic acid, and water in *N*,*N*-dimethylformamide at 80 °C for 1 day. ^1^H NMR of digested Hf-TP-OH indicated successful incorporation of the H_2_TP-OH ligand in the framework (Figure S1). Finally, Hf-TP-OH was treated with 4-nitrophenyl chloroformate followed by SN38 to afford Hf-TP-SN.

**Figure 1:**
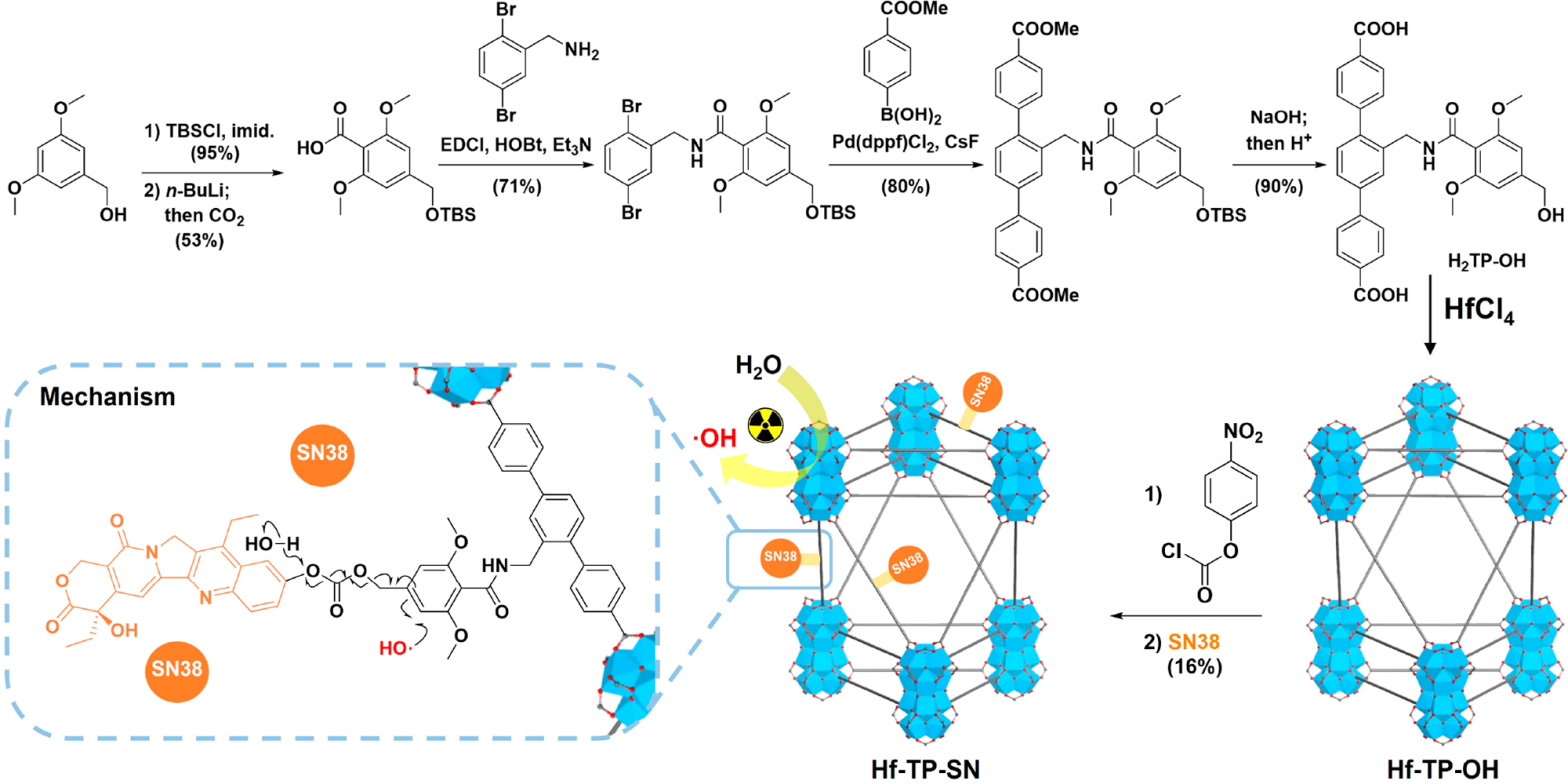
Synthesis of Hf-TP-OH nMOF and its postsynthetic modification with SN38 to afford Hf-TP-SN nMOF along with the proposed mechanism for X-ray triggered release of SN38 from Hf-TP-SN.

Hf-TP-OH and Hf-TP-SN displayed a nanoplate morphology with a diameter of ∼70 nm and a thickness of ∼10 nm, as determined by transmission electron microscopy (TEM, Figure 2a and c). The high-resolution TEM (HRTEM) image and its fast Fourier transform (FFT) pattern of Hf-TP-OH demonstrated crystallinity and 6-fold symmetry, consistent with the projection of the Hf_12_-TP (TP = terphenyldicarboxylate) structure along the vertical direction (Figure 2b). The number-averaged sizes of Hf-TP-OH and Hf-TP-SN determined by dynamic light scattering (DLS) were 125 ± 4 and 124 ± 2 nm, respectively, while their ζ-potentials were −11.2 ± 1.2 and −10.3 ± 0.4 mV, respectively (Figures 2d and S1).

**Figure 2:**
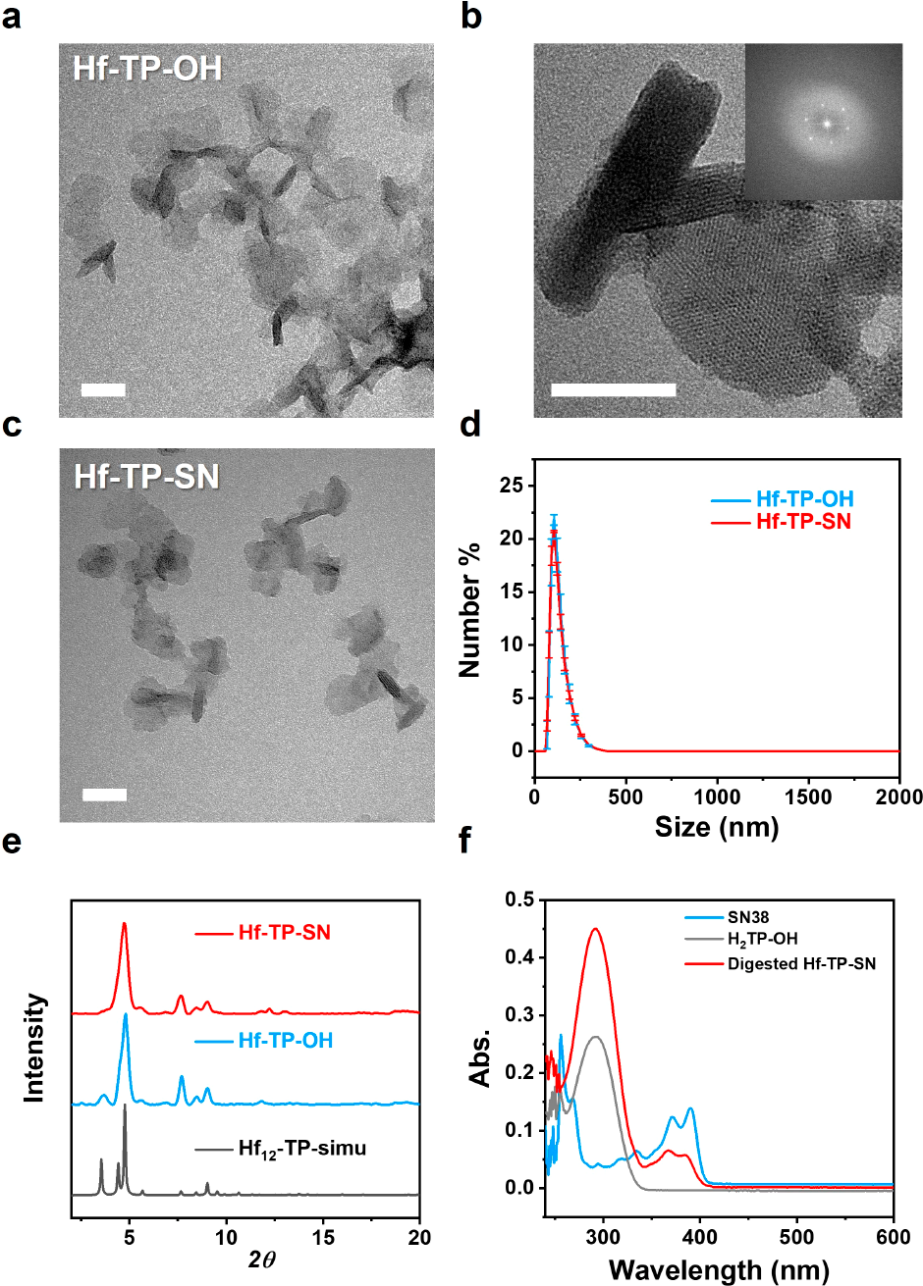
(a) TEM image and (b) HRTEM image and FFT pattern (inset) of Hf-TP-OH. (c) TEM image of Hf-TP-SN. Scale bar: 50 nm. (d) Number-averaged sizes of Hf-TP-OH and Hf-TP-SN. (e) PXRD patterns of Hf-TP-OH, Hf-TP-SN and simulated pattern for Hf_12_-TP MOF. (f) UV–vis spectra of SN38, H_2_TP-OH, and digested Hf-TP-SN in dimethyl sulfoxide.

Powder X-ray diffraction (PXRD) studies confirmed that Hf-TP-SN and Hf-TP-OH adopted the same structure as Hf_12_-TP MOF consisting of Hf_12_(μ_3_-O)_8_(μ_3_-OH)_8_(μ_2_-OH)_6_ SBUs and TP ligands in a *hcp* topology (Figure 2e).^44^ UV–vis spectroscopic analysis of digested Hf-TP-SN indicated that ∼16% of the TP-OH ligands were conjugated with SN38 (Figures 2f and S2). Liquid chromatography–mass spectrometry (LC-MS) analysis indicated the trapping of 2.6% free SN38 (relative to total SN38) in the pores after digesting Hf-TP-SN using NaHCO_3_^45^ and extraction with ethyl acetate (Figure S3). After the Hf content was determined by inductively coupled plasma mass spectrometry (ICP-MS), the formula of Hf-TP-SN was determined as Hf_12_(μ_3_-O)_8_(μ_3_-OH)_8_(μ_2_-OH)_6_(TP-SN)_1.08_(TP-OH)_5.66_(OH)_4.52_(H_2_O)_4.52_(SN38)_0.03_. Lastly, PXRD studies showed that Hf-TP-SN retained its crystallinity after incubation in 1× PBS (pH 7.4) for 24 h, while the stability of Hf-TP-SN after irradiation and long-term storage was confirmed by TEM, PXRD, and DLS (Figures S4 and S5).

A molecular counterpart, Me_2_TP-SN, was synthesized to support the postsynthetic modification and to examine the cytotoxicity of the prodrug (Schemes S2 and S3). Because of the low aqueous solubility of Me_2_TP-SN, we synthesized MeO-SN from 3,5-dimethoxylbenzyl alcohol and used it as a molecular control (Scheme S4). We first evaluated ROS generation by 2′,7′-dichlorodihydrofluorescein (DCFH) assay.^46^ The total ROS signals in the PBS, Hf-TP-OH, and Hf-TP-SN groups all increased linearly with X-ray doses. The relative enhancements of Hf-TP-OH and Hf-TP-SN over PBS were 47% and 19%, respectively (Figure 3a). Aminophenyl fluorescein (APF) assay showed that Hf-TP-OH and Hf-TP-SN enhanced ·OH generation by 96% and 59%, respectively, over PBS (Figure 3b). The reduced ROS and hydroxyl radical signals from Hf-TP-SN are likely due to the consumption of ·OH by the 3,5-dimethoxylbenzyl carbonate linkage to release SN38.

**Figure 3:**
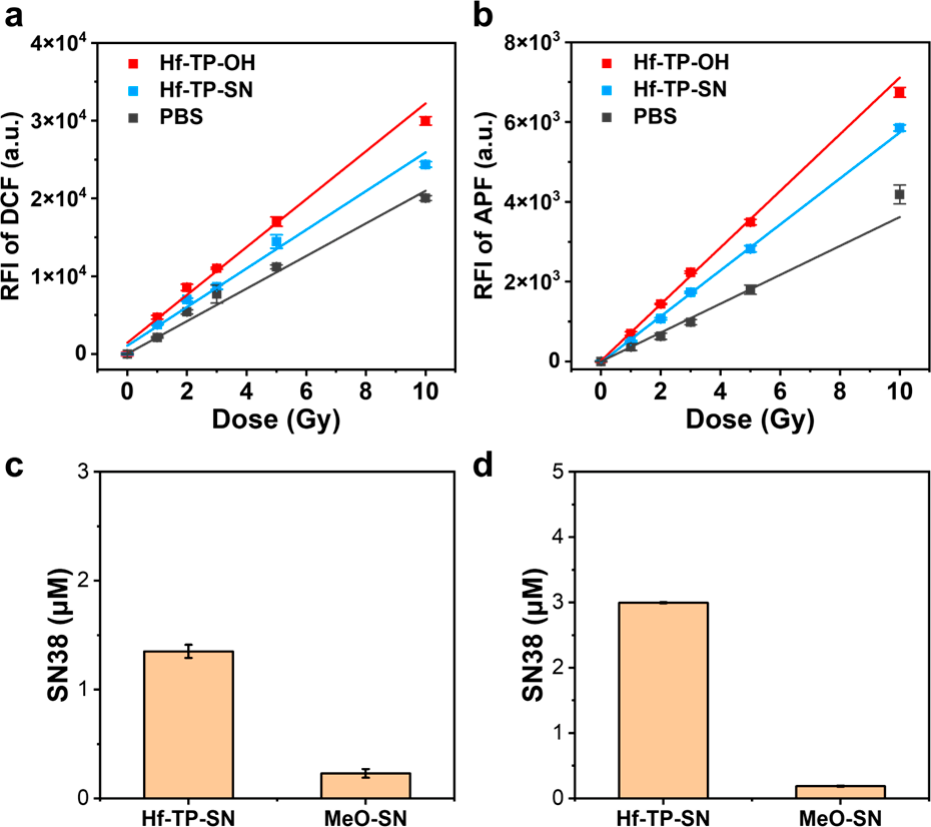
Relative fluorescence intensity (RFI) of (a) DCF and (b) APF indicating total ROS signals and hydroxyl radical signals, respectively, from PBS, Hf-TP-OH, and Hf-TP-SN with different X-ray doses, *n* = 6. Hf concentration was 40 μM. SN38 released from MeO-SN or Hf-TP-SN after 10 Gy X-ray irradiation (c) or reacting with ·OH generated by the Fenton reaction (d). Starting MeO-SN or Hf-TP-SN concentration was 100 μM.

High performance-liquid chromatography analyses showed that Hf-TP-SN released ∼1.35% of total SN38 after 10 Gy X-ray irradiation, which was 5-fold higher than SN38 released from MeO-SN under identical conditions (Figures 3c and S6). X-ray triggered release of SN38 was confirmed in a time-dependent release study (Figure S7) and using ·OH generated by the Fenton reaction (Figures 3d and S6).^47^ Hf-TP-SN showed 14.8-fold higher SN38 release than MeO-SN under the Fenton reaction. These results showed that upon X-ray irradiation electron-dense Hf_12_ SBUs serve as radiosensitizers to enhance ·OH generation for the triggered release of SN38 via hydroxylation of the 3,5-dimethoxylbenzyl carbonate followed by 1,4-elimination (Figure 1).

H_2_TP-OH ligand and Hf-TP-OH nMOF did not show obvious cytotoxicity to CT26 colon carcinoma cells at a TP concentration of 100 μM by MTS assay (Figure S8), indicating their nontoxic nature. On the other hand, clonogenic assay showed that Hf-TP-OH possessed a strong radiosensitizing property with a dose modifying ratio at 10% survival fraction (DMR_10%_) of 1.255, 1.036, and 1.333 on CT26, 4T1, and MC38 cells, respectively (Figures 4a–c and S9–S11), due to enhanced ·OH generation via radiosensitization (Figure S12).

**Figure 4:**
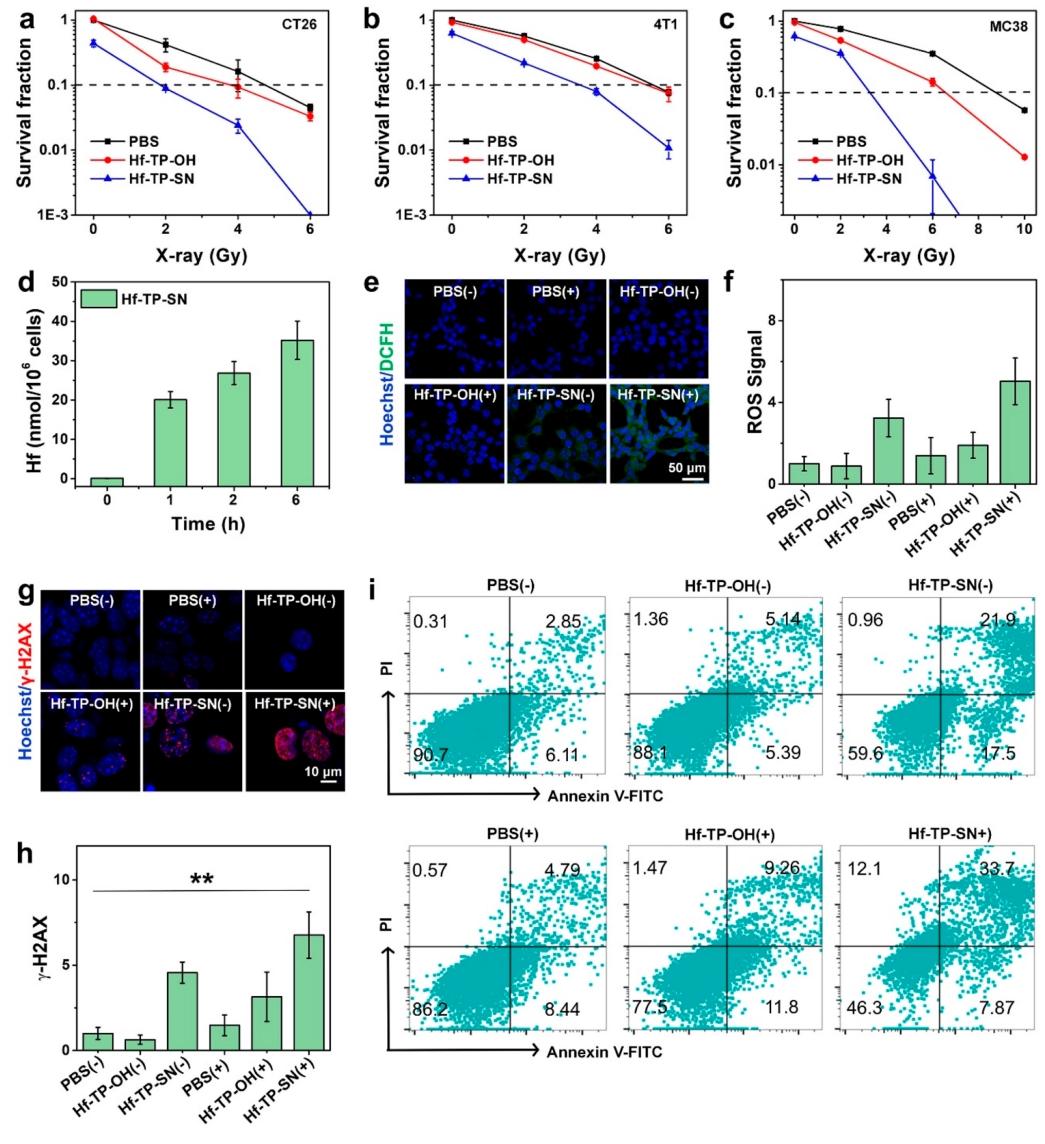
Survival fractions of (a) CT26, (b) 4T1, and (c) MC38 cells after incubation with PBS, Hf-TP-OH, or Hf-TP-SN under different X-ray doses. (d) Time-dependent cellular uptake of Hf-TP-SN quantified by ICP-MS (*n* = 3). (e) CLSM images of CT26 cells stained by DCFH-DA (green) and Hoechst 33342 (blue, cell nucleus) for ROS detection (scale bar: 50 μm). (f) Relative intracellular ROS signals (*n* = 3). (g) CLSM images of CT26 cells stained by γ-H2AX (scale bar: 10 μm). (h) Relative γ-H2AX^+^ cells (*n* = 3). (i) Representative flow cytometry dot plots showing cell apoptosis/death stained with FITC-annexin-V and PI in different treatment groups. The X-ray dose is 3 Gy in (e)–(i).

The cytotoxicities of SN38 and Me_2_TP-SN38 were also evaluated. While SN38 showed a half-maximal inhibitory concentration (IC_50_) of 0.584 μM,^33^ Me_2_TP-SN38 had much lower cytotoxicity with an IC_50_ of 13.5 μM (Figure S13). Hf-TP-SN showed efficient and time-dependent cellular uptake by CT26 cells through the clathrin-dependent pathway and macropinocytosis (Figures 4d and S14).^48,49^ To confirm X-ray triggered release of SN38, we detected the cytotoxicity of Hf-TP-SN on CT26 cells with varying doses of X-ray by clonogenic assay [(+) and (-) denote with and without radiation, respectively]. Hf-TP-SN showed a significantly increased DMR_10%_ of 2.566, 1.575, and 2.663 over Hf-TP-OH (1.255, 1.036, and 1.333) on CT26, 4T1 and MC38 cells, respectively, likely due to the combined chemoradiotherapeutic effects of the released SN38 and X-ray irradiation (Figure 4a–c). Slight cytotoxicity of Hf-TP-SN at 0 Gy likely resulted from the rapid release of entrapped SN38. Interestingly, confocal laser scanning microscopy (CLSM) and flow cytometry studies showed that Hf-TP-OH(+) and Hf-TP-SN(+) exhibited higher intracellular ROS signals than that of PBS(+) at an X-ray dose of 3 Gy (Figures 4e,f and S15; Table S1). The stronger ROS signal in the Hf-TP-SN(+) group likely resulted from the oxidative pressure of the released SN38 on the cells and the radiosensitizing effect of Hf_12_ SBUs.^50^

We next investigated DNA double strand breaks (DSBs) in CT26 cells via detection of γ-H2AX, a phosphorylated protein biomarker for DNA DSBs (Figure 4g and h).^51^ PBS(+) induced a small amount of red γ-H2AX fluorescence due to X-ray’s ability to cause DNA damage.^52^ More pronounced DSBs were observed in Hf-TP-OH(+) and Hf-TP-SN(+) groups, while no fluorescence was observed in the Hf-TP-OH(-) group, which supports potent radiosensitization by Hf-nMOFs. Hf-TP-SN(-) also showed an enhanced γ-H2AX signal over the PBS control, likely due to the entrapped SN38 in Hf-TP-SN. Free SN38 inhibits the nuclear enzyme topoisomerase I during DNA replication, leading to DSBs.^42,43^

Cell death pathways were evaluated with annexin V alexa fluor 488 and a propidium iodide (PI) cell apoptosis kit. More than 85% cells remained healthy in Hf-TP-OH(-) group, confirming negligible cytotoxicity of Hf-TP-OH (Figures 4i and S16). While Hf-TP-OH(+) and Hf-TP-SN(-) groups showed 77.5% and 59.6% healthy cells, respectively, Hf-TP-SN(+) treatment significantly reduced the healthy cell percentage to 46.3%. This result suggests synergistic therapeutic effects of Hf-TP-SN(+) due to the radiosensitizing effect of the nMOF and the potent chemotherapeutic effect of the released SN38 (Figure S17).

We established subcutaneous CT26 and 4T1 tumor models to assess the in vivo anticancer efficacy of Hf-TP-SN(+). CT26 or 4T1 tumor-bearing mice were intratumorally injected with PBS, irinotecan (a prodrug of SN38, 0.047 μmol),^34^ Hf-TP-OH (0.5 μmol Hf), or Hf-TP-SN (0.5 μmol Hf and 0.047 μmol SN38). Six to eight hours later, the tumors were irradiated with 2 Gy X-ray. X-ray irradiation was repeated on two consecutive days (6 Gy total, Figure S18). Tumor volumes and mouse body weights were monitored daily. While PBS(+) moderately inhibited the growth of CT26 and 4T1 tumors with tumor growth inhibition indices (TGIs) of 0.468 and 0.328, respectively, Hf-TP-OH(+) increased the TGIs to 0.869 and 0.721 for CT26 and 4T1 tumors, respectively. Hf-TP-SN(+) potently inhibited CT26 and 4T1 tumors with TGIs of 0.965 and 0.889, respectively. Hf-TP-SN(+) completely eradicated CT26 tumors in 40% mice. In contrast, Hf-TP-SN(-) treatment modestly inhibited CT26 and 4T1 tumors with TGIs of 0.362 and 0.162, respectively (Figures 5a–f, S19, and S20; Tables S2 and S3). The impressive in vivo therapeutic effects of Hf-TP-SN(+) resulted from synergistic actions of nMOF-mediated radiosensitization and X-ray triggered release of SN38 from Hf-TP-SN. The mice in all treatment groups showed steady body weights (Figure S21), suggesting the lack of general toxicity. Furthermore, histologies of the hearts, livers, spleens, lungs, and kidneys of treated mice did not show any abnormalities (Figure S22), supporting the safety of Hf-TP-SN(+) treatment in mice.

**Figure 5:**
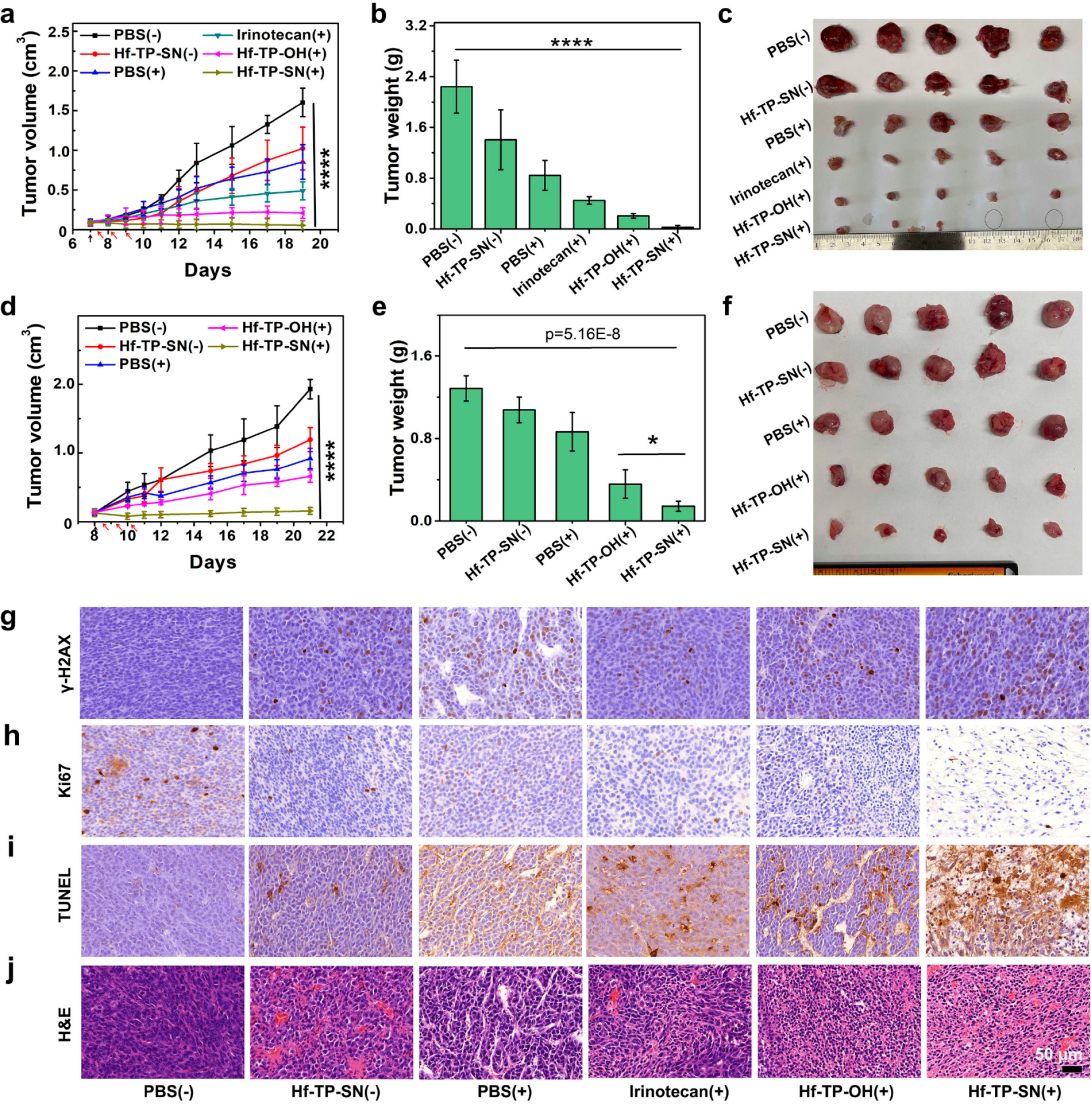
(a) Growth curves, (b) end point weights (day 19), and (c) photos of CT26 tumors in BALB/c mice after treatment with PBS, irinotecan, Hf-TP-OH, or Hf-TP-SN followed by X-ray irradiation. (d) Growth curves, (e) end point weights (day 21), and (f) photos of 4T1 tumors in BALB/c mice after different treatments. *n* = 5. Black arrows indicate nMOF injection whereas red arrows indicate irradiation. (g) γ-H2AX, (h) Ki67, (i) TUNEL and (j) H&E staining of excised CT26 tumors (scale bar: 50 μm).

One day after the last X-ray irradiation, tumor tissues were processed to evaluate pathological changes via γ-H2AX, Ki67, and terminal deoxynucleotidyl transferase mediated dUTP-biotin nick end labeling (TUNEL), and hematoxylin and eosin (H&E) staining. Hf-TP-SN(+) treatment increased the expression of γ-H2AX (Figures 5g and S23), reduced cell proliferation as indicated by a lower Ki67 signal (Figures 5h and S24), and increased cell apoptosis as determined via TUNEL staining (Figures 5i and S25). H&E staining showed distinctive cellular damage in the tumors treated with Hf-TP-OH(+) and Hf-TP-SN(+); minimal cellular damage was observed in PBS(+) and irinotecan(+) groups (Figure 5j).

In summary, we designed a Hf-TP-SN nMOF with an X-ray triggerable SN38 prodrug for synergistic radiotherapy and chemotherapy. Hf-TP-SN contains terphenyl ligands conjugated with SN38 via a carbonate bond. Upon X-ray irradiation, Hf_12_-SBUs served as radiosensitizers to enhance ·OH generation and increase SN38 release from Hf-TP-SN. Hf-TP-SN not only enhanced the radiotherapeutic efficacy but also achieved chemotherapeutic effects through triggered release of SN38. Such a chemoradiotherapy strategy effectively reduces the radiation dose required for tumor regression and minimizes the side effects of chemoradiotherapy via the burst release of SN38 inside cancer cells. This work highlights the potential of nMOFs in multimodality cancer treatment via on-demand, triggered release of therapeutic agents.

## Supporting Information Available

Materials and methods, synthesis and characterization, total ROS generation, in vitro and in vivo experiments (PDF)
