# Supplementary material for: Nanoscale Metal–Organic Framework with an X-ray Triggerable Prodrug for Synergistic Radiotherapy and Chemotherapy
Source: J Am Chem Soc. 2023 Aug 15;145(34):18698–704. doi: 10.1021/jacs.3c04602 (PMC10472429; doi:10.1021/jacs.3c04602)
Supplement: Supplementary file 1 — ja3c04602_si_001.pdf [file ja3c04602_si_001.pdf]

Supporting Information for

Nanoscale Metal-Organic Framework with an X-ray Triggerable Prodrug for Synergistic Radiotherapy and Chemotherapy

Ziwan Xu,[1,‡] Wenyao Zhen,[1,2,‡] Caroline McCleary,[1] Taokun Luo,[1] Xiaomin Jiang,[1] Cheng Peng,[1] Ralph R. Weichselbaum,[2] and Wenbin Lin[1,2,*]

[1]Department of Chemistry, The University of Chicago, Chicago, Illinois 60637, United States

[2]Department of Radiation and Cellular Oncology and Ludwig Center for Metastasis Research, The University of Chicago, Chicago, Illinois 60637, United States

Email: wenbinlin@uchicago.edu

Table of Contents

S1. Materials and Methods 1

S2. Synthesis of Organic Ligands and Molecular Prodrugs 2

S3. Hf-TP-OH Synthesis and Post-synthetic modification 6

S4. Characterization 8

S5. In Vitro Experiments 13

S6. In Vivo Experiments 20

S7. NMR spectra 26

S8. References 37

S1. Materials and Methods

All starting chemicals for the synthesis of nanoscale metal-organic frameworks (nMOFs) were purchased from Sigma-Aldrich or Fisher (USA) and used directly without purification. Phosphate buffered saline (PBS) for cell culture and RPMI-1640 medium were purchased from Corning, USA. Trypsin-EDTA solution and HyClone penicillin-streptomycin 100X solution were purchased from Cytiva USA. 2',7'-dichlorodihydrofluorescein diacetate (DCFH-DA), dead cell apoptosis kit with annexin V Alexa Fluor 488 & propidium iodide (PI), hydroxyphenyl fluorescein (HPF), and aminophenyl fluorescein (APF) were purchased from Invitrogen. Chlorpromazine was purchased from Thermo Scientific. Nystatin, and rottlerin were purchased from MedChemExpress. CellTiter 96® AQueous One Solution Cell Proliferation Assay (MTS) was purchased from Promega, USA. Phospho-histone H2A.X (Ser139) (γ-H2AX) rabbit monoclonal antibody and anti-rabbit IgG (H+L), F(ab')2 Fragment (Alexa Fluor® 488 Conjugate) were purchased from Cell Signaling Technology. Murine colorectal carcinoma CT26 cells were obtained from the American Type Culture Collection (ATCC, Rockville, MD). MC38 and 4T1 cells were obtained from Dr. Weichselbaum and Prof. Kron at the University of Chicago, respectively.

Transmission electron microscopy (TEM) was carried out on an FEI Spirit 120kV LaB6 Electron Microscope. Powder X-ray diffraction (PXRD) data were collected on a Bruker D8 Venture diffractometer using a Cu Kα radiation source (λ = 1.54178 Å) and processed with PowderX software, or collected on a Small Angle X-ray Scattering instrument, GANESHA. UV-Vis spectra were collected using a Shimadzu UV-2600 UV-Vis spectrophotometer. Fluorescence emission and excitation spectra were obtained using a Shimadzu RF-5301PC spectrofluorophotometer. Dynamic light scattering (DLS) and ζ-potential measurements were performed on a Malvern Zetasizer Nano ZS instrument. Inductively coupled plasma-mass spectrometry (ICP-MS) data were collected using an Agilent 7700x ICP-MS and analyzed using an ICP-MS Mass Hunter version 4.6 C.01.06. Samples were diluted in a 2% HNO_3 matrix and analyzed with ^{159}Tb as internal standards against a 10-point standard curve between 1 ppb and 500 ppb (R>0.999 for ^{178}Hf and ^{232}Th). Data collection was performed in Spectrum Mode with triplicates per sample and 100 sweeps per replicate. ^{1}H NMR spectra were recorded on a Bruker Ultrashield Plus spectrometer at 400 MHz and referenced to the proton resonance resulting from incomplete deuteration of $CDCl_3$ (δ = 7.26) or DMSO-d_6 (δ = 2.50). SN38 concentration was quantified using HPLC (Shimadzu Prominence UFLC/HPLC system with LC-20AT pump, Shimadzu Corporation, Japan) or LC-MS (Agilent 6540, Agilent Technologies, USA).

For test tube and in vitro X-ray irradiation experiments, an RT250 orthovoltage X-ray machine (Philips, USA) with fixed setting at 250 kVp, 15 mA and a built-in 1 mm Cu filter was used. For animal irradiation and computerized tomography (CT) scans, an X-RAD 225 image-guided biological irradiator (Precision X-ray Inc., USA) was used with voltage at 225 kVp, current at 13 mA, a 0.3 mm Cu filter, and a 15 mm collimator. The X-ray dose rate of X-RAD 225 was 0.04167 Gy/second. The X-ray dosimetry of both instruments was calibrated with an ionization chamber regularly by the Department of Radiation Oncology at the University of Chicago. Flow cytometry data were collected on an LSR-Fortessa 4-15 HTS (BD Biosciences, USA) at the Cytometry and Antibody Technology Facility at the University of Chicago and analyzed by FlowJo software (Tree Star, USA). Confocal laser scanning microscopy (CLSM) images were collected on a Leica Stellaris 8 laser scanning microscope at the Integrated Light Microscopy Facility at the University of Chicago, and analysis was done with Image J software (NIH, USA). Live cell images were recorded and analyzed by IncuCyte S3 (Essen

BioScience) at Cellular Screening Center at the University of Chicago. The histological slides were scanned on a CRi Pannoramic SCAN 40x whole slide scanner by Integrated Light Microscopy Core at the University of Chicago and analyzed with the QuPath-0.2.3 software. The absorbance and fluorescence intensities from well plates were read by a BioTek Synergy HTX microplate reader.

P value was calculated by student's two-tailed t test, *, p<0.05, **, p<0.01, ***, p<0.001, and ****, p<0.0001 were used in all figures to show the statistical significance.

S2. Synthesis of Organic Ligands and Molecular Prodrugs

S2.1 Synthesis of H_2TP-OH ligand

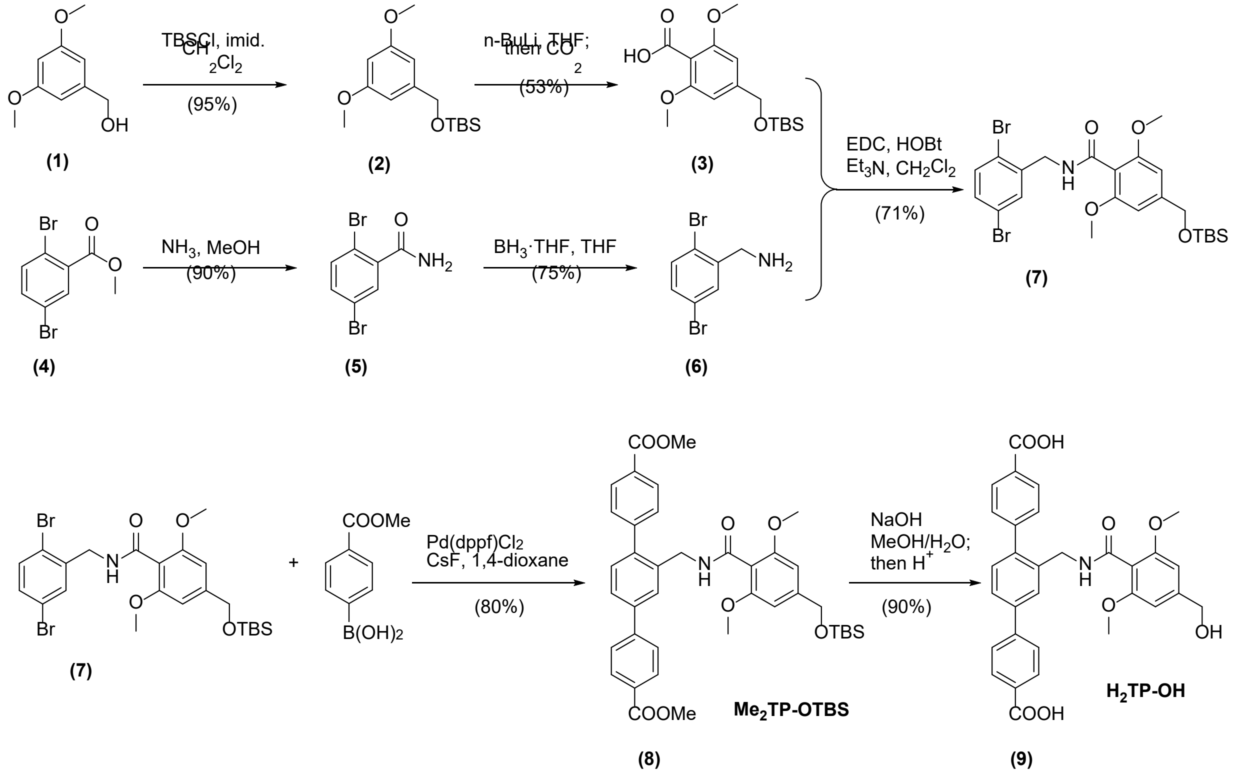

Scheme S1. Synthetic route for H_2TP-OH (**9**).

4-(((*tert*-Butyldimethylsilyl)oxy)methyl)-2,6-dimethoxybenzoic acid (3). A solution of imidazole (6.08 g, 0.089 mol) and 3,5-dimethoxybenzyl alcohol (**1**, 5 g, 0.030 mol) in dry dichloromethane (DCM, 60 mL) was combined with tert-butyldimethylsilyl chloride (TBSCl, 6.74 g, 0.045 mol) and stirred at room temperature (RT) for 2 hours. Excess TBSCl was quenched by 2 mL methanol (MeOH); the resulting solution was condensed via rotary evaporation and then purified by flash silica gel column chromatography to give *tert*-butyl((3,5-dimethoxybenzyl)oxy)dimethylsilane (**2**, 8 g, 0.028 mol, 95%). ^{1}H NMR (400 MHz, Chloroform-*d*) δ 6.52-6.47 (m, 2H), 6.34 (s, 1H), 4.69 (s, 2H), 3.79 (s, 6H), 0.95 (s, 9H), 0.10 (s, 6H); ^{13}C NMR (101 MHz, $CDCl_3$) δ 160.91, 144.23, 103.85, 99.05, 64.99, 55.42, 26.09, 18.57, -5.11. HR-MS (ESI, positive mode): *m/z* calc'd for $C_{15}H_{27}O_3Si$ $[M+H]^+$: 283.1729, found 283.1727.

Compound **2** (10 g, 0.035 mol) was dissolved in dry tetrahydrofuran (THF, 100 mL) and cooled to 0 °C under N_2, to which 1.6 M n-butyllithium in hexanes (22.2 mL, 0.035 mol) was added dropwise;

the resulting mixture was then stirred at RT for 1 hour. Dry ice (10 g, 0.227 mol) was added afterwards, and the reaction mixture was allowed to stand at RT overnight. The resulting solution was condensed via rotary evaporation and water (50 mL) was added to the residue, which was extracted with ethyl acetate (EA, 20 mL) twice. The aqueous layer was carefully acidified with 1M hydrochloric acid (dropwise) until the pH reached about 3. The white precipitate was separated via filtration, washed with water, dried under vacuum, and purified by column chromatography to give **3** (6.1 g, 0.019 mol, 53%). ^{1}H NMR (400 MHz, Chloroform-*d*) δ 6.60 (s, 2H), 4.74 (s, 2H), 3.88 (s, 6H), 0.96 (s, 8H), 0.12 (s, 6H); ^{13}C NMR (101 MHz, $CDCl_3$) δ 168.75, 158.57, 146.94, 109.33, 101.59, 64.73, 56.35, 26.01, 18.51, -5.12. HR-MS (ESI, positive mode): *m/z* calc'd for $C_{16}H_{27}O_5Si$ $[M+H]^+$: 327.1628, found 327.1695.

2,5-Dibromobenzyl amine (6). 2,5-Dibromobenzyl amine (**6**) was prepared based on reported procedures with modification in the second step.[1] To a solution of 7M ammonia (NH_3) in MeOH (100 mL, 0.70 mol) was added methyl 2,5-dibromobenzoate (**4**, 10.0 g, 0.034 mol) at RT; the resulting dissolved solution was evenly separated into five 20-mL vials, sealed, and stirred for 3 days. The white precipitate formed was separated and collected via filtration, dispersed in hexanes (100 mL), recollected via filtration, and dried under vacuum to produce 2,5-dibromobenzamide (**5**, 8.58 g, 0.031 mol, 90%). ^{1}H NMR (400 MHz, Chloroform-*d*) δ 7.78 (d, *J* = 2.4 Hz, 1H), 7.48 (d, *J* = 8.5 Hz, 1H), 7.42 (dd, *J* = 8.5, 2.4 Hz, 1H), 6.02 (d, *J* = 63.0 Hz, 2H); ^{13}C NMR (101 MHz, $CDCl_3$) δ 167.63, 138.29, 135.14, 134.84, 133.00, 121.79, 117.98. HR-MS (ESI, positive mode): *m/z* calc'd for $C_7H_6Br_2NO$ $[M+H]^+$: 279.8796, found 279.8790.

1M borane-tetrahydrofuran complex (BH_3 in THF) was added in excess (35 mL, 0.035 mol) to **5** (4.11 g, 0.0144 mol); the resulting mixture was stirred at reflux for 24 hours and then quenched with concentrated HCl (3.5 mL). The solution was then refluxed for 2 more hours and cooled to RT. The suspension was filtered to obtain the white solid, which was then dissolved in water and basified to pH~10 by addition of sat. sodium carbonate (Na_2CO_3) while the solution was stirring. The reaction mixture was extracted with EA (30 mL) 3 times. The combined organic phase was dried over sodium sulfate (Na_2SO_4), filtered, and evaporated to give **6** (2.91 g, 0.011 mol, 74.6%). ^{1}H NMR (400 MHz, Chloroform-*d*) δ 7.55 (d, *J* = 2.6 Hz, 1H), 7.39 (d, *J* = 8.5 Hz, 1H), 7.24 (dd, *J* = 8.4, 2.4 Hz, 1H), 3.88 (s, 2H); ^{13}C NMR (101 MHz, $CDCl_3$) δ 144.34, 134.22, 131.99, 131.45, 122.07, 121.76, 46.65. HR-MS (ESI, positive mode): *m/z* calc'd for $C_7H_8Br_2N$ $[M+H]^+$: 265.9003, found 265.8999.

4-(((*tert*-Butyldimethylsilyl)oxy)methyl)-N-(2,5-dibromobenzyl)-2,6-dimethoxybenzamide (7). Compound **6** (1 g, 3.77 mmol), **3** (1.23 g, 3.77 mmol), EDCI (1.536 g, 8.0 mmol), HOBt (0.6486 g, 4.8 mmol), and triethylamine (TEA, 1.3675 mL) were combined in 75 mL dry DCM under Ar. The resulting mixture was stirred at RT for 12 hours before 10 mL H_2O was added and the mixture was extracted by DCM (3x20 mL); the combined organic phase was then dried over Na_2SO_4, filtered, evaporated, and separated via column chromatography to give **7** (1.534 g, 2.68 mmol, 71%). ^{1}H NMR (400 MHz, Chloroform-*d*) δ 7.83 (dd, *J* = 2.4, 0.9 Hz, 1H), 7.39 (dd, *J* = 8.4, 0.9 Hz, 1H), 7.24 (d, *J* = 2.4 Hz, 1H), 6.58 (d, *J* = 1.0 Hz, 2H), 6.17 (t, *J* = 6.5 Hz, 1H), 4.73 (d, *J* = 1.0 Hz, 2H), 4.68 (d, *J* = 6.4 Hz, 2H), 3.88 (d, *J* = 0.9 Hz, 6H), 0.96 (d, *J* = 0.9 Hz, 9H), 0.11 (d, *J* = 0.9 Hz, 6H); ^{13}C NMR (101 MHz, $CDCl_3$) δ 166.22, 157.58, 145.50, 139.87, 133.93, 131.86, 121.83, 121.53, 113.82, 101.42, 64.90, 56.04, 43.48, 26.03, 18.53, -5.09. HR-MS (ESI, positive mode): *m/z* calc'd for $C_{23}H_{32}Br_2NO_4Si$ $[M+H]^+$: 574.0447, found 574.0463.

Dimethyl 2''-((4-(((*tert*-butyldimethylsilyl)oxy)methyl)-2,6-dimethoxybenzamido)methyl)-[1,1':4',1'':4'',1'''-quaterphenyl]4,4'''-dicarboxylate (8). To **7** (1 g, 0.0017 mol) was added (4-(methoxycarbonyl)phenyl)boronic acid (0.942 g, 0.0052 mol), CsF (1.591 g, 0.0105 mol), and Pd(dppf)Cl_2 (0.128 g, 0.00017 mol); the solids were collected in a Schlenk flask and put under vacuum. Degassed dioxane (45 mL) was added to the flask; the resulting solution was stirred at 90 °C for 3 days, after which the solution was allowed to cool to RT, filtered through celite, rid of dioxane via rotary evaporation, dried under vacuum and purified via column chromatography to yield **8** (0.95 g, 1.4 mmol, 80%). ^{1}H NMR (400 MHz, Chloroform-*d*) δ 8.15 – 8.10 (m, 4H), 7.99 (d, *J* = 1.9 Hz, 1H), 7.78 – 7.72 (m, 2H), 7.60 (dd, *J* = 7.9, 1.9 Hz, 1H), 7.51 – 7.45 (m, 2H), 7.36 (d, *J* = 7.9 Hz, 1H), 6.53 (s, 2H), 5.98 (t, *J* = 6.0 Hz, 1H), 4.70 (s, 2H), 4.68 (d, *J* = 6.0 Hz, 2H), 3.96 (d, *J* = 2.8 Hz, 6H), 3.71 (s, 6H), 0.94 (s, 9H), 0.10 (s, 6H); ^{13}C NMR (101 MHz, $CDCl_3$) δ 167.11, 167.01, 166.13, 157.55, 145.34, 145.30, 145.02, 140.23, 140.05, 136.57, 130.49, 130.24, 129.85, 129.48, 129.39, 129.27, 127.30, 127.20, 126.09, 114.05, 101.43, 64.88, 56.00, 52.37, 52.33, 41.48, 26.02, 18.52, -5.10. HR-MS (ESI, positive mode): *m/z* calc'd for $C_{39}H_{46}NO_8Si$ $[M+H]^+$: 684.2993, found 684.3101.

2'-((4-(Hydroxymethyl)-2,6-dimethoxybenzamido)methyl)-[1,1':4',1''-terphenyl]-4,4''-dicarboxylic acid (H_2TP-OH, 9). To **8** (0.6911 g, 1.01 mmol) was added NaOH (4.04 g, 101 mmol, 50 eq. per ester group) and MeOH and H_2O in a 1:1 ratio (20 mL: 20 mL). The resulting mixture was stirred at 40 °C for 1 day; it was then allowed to cool to RT before being acidified (while being stirred) to pH~1-2 by addition of concentrated HCl; the acidic solution was left to stir for 2 hours to ensure the completion of the acid-base reaction. The resulting solution was filtered, washed with H_2O and dried under vacuum to produce H_2TP-OH (**9**, 0.493 g, 0.91 mmol, 90%).^{1}H NMR (400 MHz, DMSO-*d*$_6$) δ 12.99 (s, 2H), 8.63 (t, *J* = 6.0 Hz, 1H), 8.11 – 8.03 (m, 4H), 7.97 (d, *J* = 2.0 Hz, 1H), 7.88 – 7.82 (m, 2H), 7.72 (dd, *J* = 8.0, 1.9 Hz, 1H), 7.63 – 7.56 (m, 2H), 7.40 (d, *J* = 8.0 Hz, 1H), 6.63 (s, 2H), 4.49 (s, 2H), 4.37 (d, *J* = 5.9 Hz, 2H), 3.63 (s, 6H). ^{13}C NMR (101 MHz, DMSO-*d*$_6$) δ 167.14, 167.12, 164.90, 156.53, 145.24, 144.26, 144.16, 139.30, 138.55, 137.25, 130.10, 130.01, 129.80, 129.70, 129.42, 129.38, 126.77, 126.34, 125.29, 114.89, 101.87, 62.90, 55.55, 25.81. HR-MS (ESI, positive mode): *m/z* calc'd for $C_{31}H_{28}NO_8$ $[M+H]^+$: 542.1815, found 542.1780.

S2.2 Synthesis of Me_2TP-SN

Dimethyl 2'-((4-(hydroxymethyl)-2,6-dimethoxybenzamido)methyl)-[1,1':4',1''-terphenyl]-4,4''-dicarboxylate (Me_2TP-OH, 10)

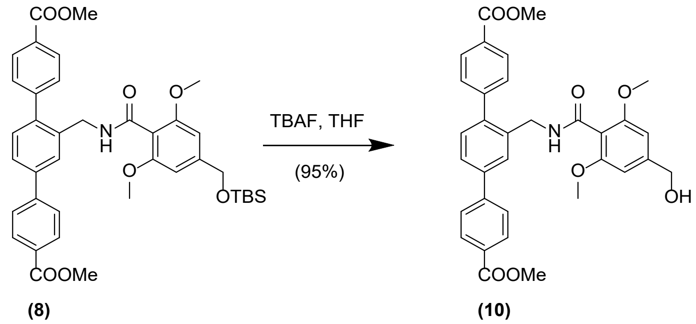

Scheme S2. Synthesis of Me_2TP-OH (**10**).

Compound **8** (500 mg, 0.730 mmol) was added to tetrabutylammonium fluoride solution in THF (1M, 5 mL) and stirred at RT for 2 hours. 10 mL saturated NH_4Cl solution was added and THF was removed via rotary evaporation. White precipitate was filtered and washed with water to produce Me_2TP-OH

(**10**, 395 mg, 0.694 mmol, 95%). ^{1}H NMR (400 MHz, Chloroform-d) δ 8.16 – 8.11 (m, 4H), 7.97 (d, J = 12.9 Hz, 1H), 7.75 (dd, J = 8.5, 2.3 Hz, 2H), 7.64 – 7.59 (m, 1H), 7.53 – 7.45 (m, 2H), 7.37 (dd, J = 7.9, 3.3 Hz, 1H), 6.56 (s, 2H), 5.96 (t, J = 6.3 Hz, 1H), 4.81 – 4.64 (m, 4H), 3.96 (d, J = 3.1 Hz, 6H), 3.73 (s, 6H). ^{1}H NMR (400 MHz, DMSO-d_6) δ 8.63 (t, *J* = 5.9 Hz, 1H), 8.09 (dd, *J* = 12.7, 7.9 Hz, 4H), 7.98 (s, 1H), 7.88 (d, *J* = 8.0 Hz, 2H), 7.74 (d, *J* = 8.1 Hz, 1H), 7.63 (d, *J* = 7.9 Hz, 2H), 7.41 (d, *J* = 7.8 Hz, 1H), 6.63 (s, 2H), 5.27 (s, 1H), 4.49 (s, 2H), 4.37 (d, *J* = 5.8 Hz, 2H), 3.90 (d, *J* = 4.4 Hz, 6H), 3.62 (s, 6H). ^{13}C NMR (101 MHz, DMSO-d_6) δ 166.08, 166.04, 164.88, 156.51, 145.24, 144.64, 144.51, 139.23, 138.43, 137.28, 130.12, 129.87, 129.59, 129.23, 128.65, 128.56, 126.93, 126.37, 125.35, 114.87, 101.87, 62.89, 55.56, 52.24, 52.21, 40.15. HR-MS (ESI, positive mode): *m/z* calc'd for $C_{33}H_{32}NO_8$ $[M+H]^+$: 570.2128, found 570.2111.

Dimethyl (*S*)-2'-((4-(((((4,11-diethyl-4-hydroxy-3,14-dioxo-3,4,12,14-tetrahydro-1H-pyrano[3',4':6,7]indolizino[1,2-b]quinolin-9-yl)oxy)carbonyl)oxy)methyl)-2,6-dimethoxybenzamido)methyl)-[1,1':4',1''-terphenyl]-4,4''-dicarboxylate (Me_2TP-SN, 12)

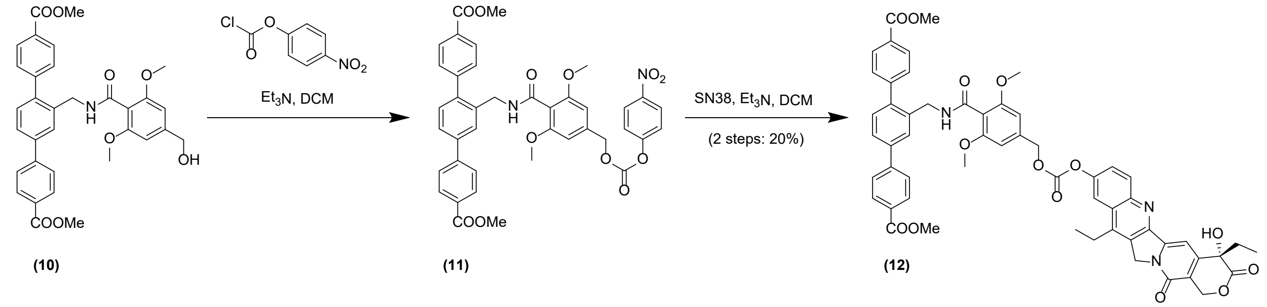

Scheme S3. Synthesis of Me_2TP-SN (**12**).

Compound **10** (500 mg, 0.878 mmol), 4-nitrophenyl chloroformate (442 mg, 2.195 mmol), TEA (366 µL, 2.634 mmol) were combined in dry DCM (30 mL) under Ar and stirred for 1 day at RT. The resulting solution was quenched with 5 mL H_2O and extracted with DCM, dried over Na_2SO_4, filtered, concentrated by rotary evaporation, and then dried completely under vacuum to produce dimethyl 2'-((2,6-dimethoxy-4-((((4-nitrophenoxy)carbonyl)oxy)methyl)benzamido)methyl)-[1,1':4',1''-terphenyl]-4,4''-dicarboxylate (**11**), which was used directly in the next step without purification.

To a solution of **11** (500 mg, 0.681 mmol) in DCM (20 mL) was added SN38 (668 mg, 1.702 mmol) and TEA (284 µL, 2.043 mmol); the resulting mixture was stirred at RT for 1 day before being washed with H_2O and extracted with DCM (3x20 mL). The organic phases were combined, dried over Na_2SO_4, filtered, concentrated by rotary evaporation, dissolved in minimal DCM, and purified via large TLC plate chromatography (5% MeOH in DCM) to give Me_2TP-SN (**12**, 134 mg, 0.136 mmol, 20%). ^{1}H NMR (400 MHz, Chloroform-*d*) δ 8.24 (d, *J* = 9.2 Hz, 1H), 8.13 (ddd, *J* = 10.2, 6.1, 2.8 Hz, 4H), 7.99 (d, *J* = 1.9 Hz, 1H), 7.91 (d, *J* = 2.6 Hz, 1H), 7.78 – 7.74 (m, 2H), 7.74 – 7.69 (m, 4H), 7.64 (s, 1H), 7.64 – 7.60 (m, 2H), 7.50 (d, *J* = 8.3 Hz, 2H), 7.42 (d, *J* = 7.9 Hz, 1H), 6.65 (s, 2H), 6.07 (t, *J* = 6.0 Hz, 1H), 5.73 (d, *J* = 16.3 Hz, 1H), 5.26 (d, *J* = 11.3 Hz, 4H), 4.77 (d, *J* = 6.1 Hz, 2H), 3.95 (d, *J* = 2.4 Hz, 6H), 3.75 (s, 6H), 3.14 (q, *J* = 7.7 Hz, 2H), 1.89 (ddt, *J* = 16.9, 14.2, 7.1 Hz, 2H), 1.38 (t, *J* = 7.7 Hz, 3H), 1.03 (t, *J* = 7.3 Hz, 3H).; ^{13}C NMR (101 MHz, $CDCl_3$) δ 174.00, 167.09, 165.58, 157.77, 157.74, 153.40, 152.33, 150.31, 149.88, 147.65, 146.90, 145.54, 145.36, 145.10, 140.67, 140.05, 139.67, 139.30, 137.72, 136.46, 132.48, 131.75, 131.00, 130.72, 130.36, 130.24, 129.99, 129.25, 129.22, 127.56, 127.52, 127.44, 127.24, 127.13, 126.99, 126.12, 124.72, 118.84, 116.13, 114.26, 104.32, 98.24, 72.89, 70.61, 66.47, 56.25,

52.34, 52.32, 49.51, 41.60, 31.75, 23.32, 14.13, 7.96. HR-MS (ESI, positive mode): *m/z* calc'd for $C_{56}H_{50}N_3O_{14}$ $[M+H]^+$: 988.3293, found 988.3273.

S2.3 Synthesis of (S)-4,11-diethyl-4-hydroxy-3,14-dioxo-3,4,12,14-tetrahydro-1H-pyrano[3',4':6,7]indolizino[1,2-b]quinolin-9-yl (3,5-dimethoxybenzyl) carbonate (MeO-SN, 14)

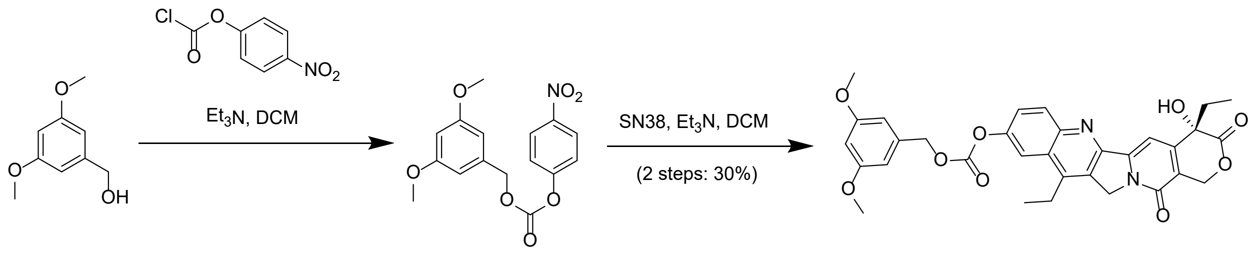

(1) (13) (14)

Scheme S4. Synthesis of MeO-SN (**14**).

Compound **1** (500 mg, 2.972 mmol), 4-nitrophenyl chloroformate (1.497 g, 7.430 mmol), TEA (1.240 mL, 8.916 mmol) were combined in dry DCM (50 mL) under Ar and stirred for 1 day at RT. The resulting solution was quenched with 5 mL H_2O and extracted with DCM, dried over Na_2SO_4, filtered, concentrated by rotary evaporation, and then dried completely under vacuum to produce dimethyl 3,5-dimethoxybenzyl (4-nitrophenyl) carbonate (**13**), which was used directly in the next step without purification.

To a solution of **13** (500 mg, 1.500 mmol) in DCM (30 mL) was added SN38 (1471 mg, 3.750 mmol) and TEA (626 µL, 4.500 mmol); the resulting mixture was stirred at RT for 1 day before being washed with H_2O and extracted with DCM (3 x 20 mL). The organic phases were combined, dried over Na_2SO_4, filtered, concentrated by rotary evaporation, dissolved in minimal DCM, and purified via column chromatography to give MeO-SN (**14**, 264 mg, 0.450 mmol, 30%). ^1H NMR (400 MHz, $CDCl_3$) δ 8.25 (d, *J* = 9.2 Hz, 1H), 7.92 (d, *J* = 2.6 Hz, 1H), 7.68 (s, 1H), 7.65 (dd, *J* = 9.2, 2.6 Hz, 1H), 6.61 (d, *J* = 2.3 Hz, 2H), 6.47 (t, *J* = 2.3 Hz, 1H), 5.74 (d, *J* = 16.4 Hz, 1H), 5.30 (d, *J* = 16.5 Hz, 1H), 5.26 (d, *J* = 3.6 Hz, 4H), 3.82 (s, 6H), 3.15 (q, *J* = 7.7 Hz, 2H), 1.88 (dq, *J* = 17.0, 7.1 Hz, 2H), 1.39 (t, *J* = 7.7 Hz, 3H), 1.03 (t, *J* = 7.4 Hz, 3H). ^{13}C NMR (101 MHz, $CDCl_3$) δ 173.93, 161.23, 157.72, 153.41, 151.89, 150.38, 150.10, 147.10, 146.56, 146.09, 136.73, 132.03, 127.60, 127.55, 125.08, 119.01, 114.27, 106.44, 100.87, 98.68, 72.90, 70.80, 66.43, 55.58, 49.57, 31.77, 23.40, 14.10, 7.96. HR-MS (ESI, positive mode): *m/z* calc'd for $C_{32}H_{31}N_2O_9$ $[M+H]^+$: 587.2030, found 587.2009.

S3. Hf-TP-OH Synthesis and Post-synthetic modification

S3.1 Synthesis of Hf-TP-OH

$HfCl_4$ and H_2TP-OH were separately dissolved in dimethylformamide (DMF) at a concentration of 2 mg/mL. 500 µL $HfCl_4$ solution and 500 µL H_2TP-OH were then combined in a 1-dram vial with the addition of 1 µL trifluoracetic acid and 5 µL water as modulators. The mixture was heated in an oven at 80 °C for 1 day, after which the white precipitate was collected by centrifugation and sequentially washed with DMF, 1% triethylamine (TEA) in ethanol (EtOH) (v/v), and EtOH to afford Hf-TP-OH in 88% yield based on H_2TP-OH.

S3.2 Synthesis of Hf-TP-SN via post-synthetic modification

10 mL Hf-TP-OH was washed with dry acetonitrile (ACN) twice and dispersed in ACN with a ligand concentration of 3.0 mM. 4-nitrophenyl chloroformate (15.1 mg, 75 μmol), and TEA (12.5 μL, 90 μmol) were then added, and the solution was stirred for 2 days to afford Hf-TP-NO_2 nMOF. The mixture was then centrifuged, washed with ACN for 3 times and redispersed in ACN before the addition of SN38 (11.8 mg, 30 μmol) and TEA (6.3 μL, 45 μmol); the solution was then stirred for 2 more days. The as-synthesized Hf-TP-SN was washed with 10% dimethyl sulfoxide (DMSO) in ACN 6 times and dispersed in EA for storage.

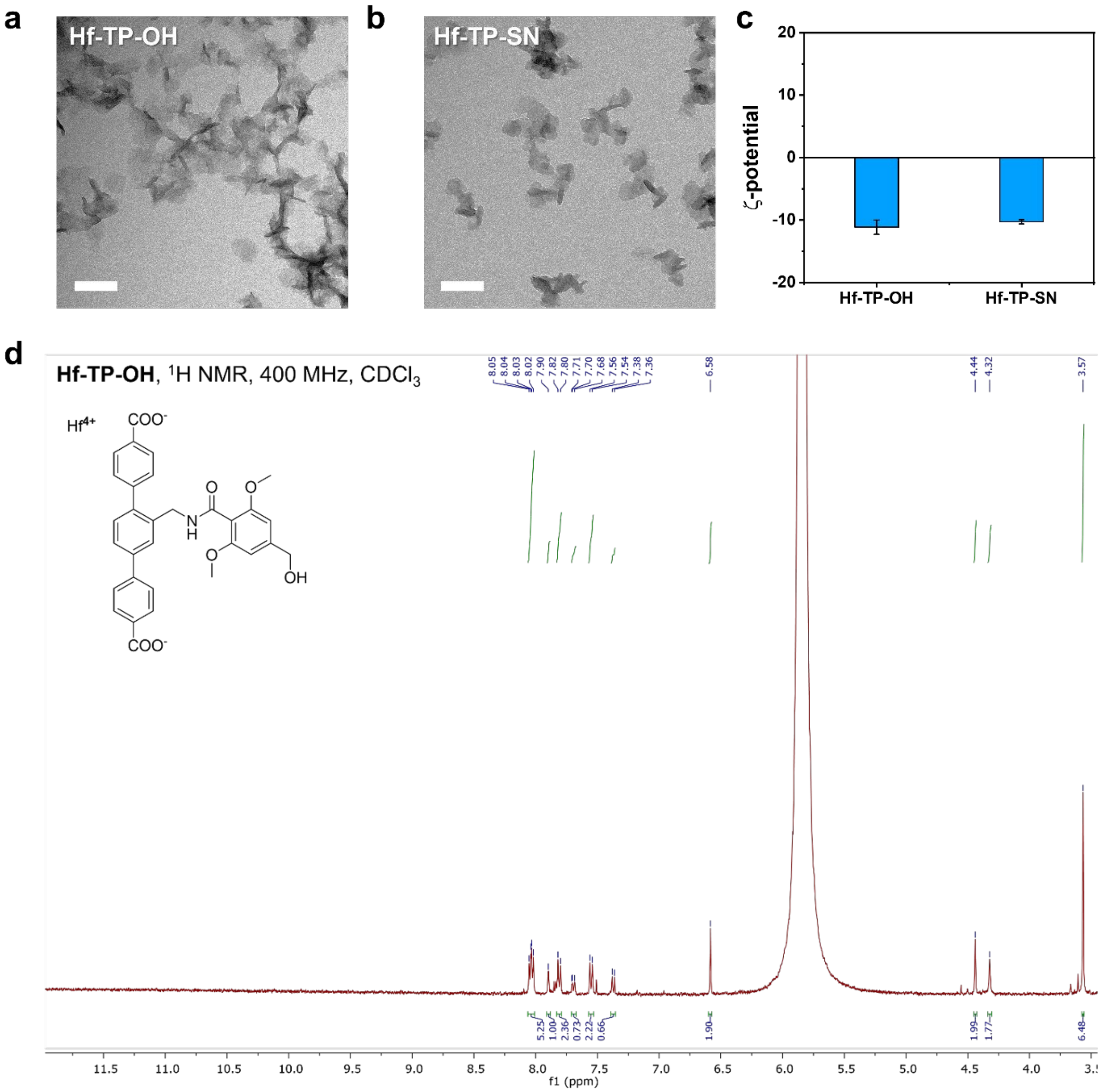

Figure S1. TEM images of (a) Hf-TP-OH and (b) Hf-TP-SN. Scale bar = 100 nm. (c) ζ -potentials of Hf-TP-OH and Hf-TP-SN in H_2O. (d) ^1H NMR spectrum of digested Hf-TP-OH.

S4. Characterization

S4.1 Digestion of Hf-MOFs for ^{1}H NMR Characterization

Hf-TP-OH was dried under vacuum overnight. 500 µL DMSO-d_6 and 50 µL D_3PO_4 were added to the resulting solid. The mixture was sonicated for 1 hour, followed by the addition of 50 µL D_2O for ^{1}H NMR analysis.

S4.2 Digestion of Hf-MOFs for UV-Vis spectroscopic measurements

50 µL Hf-TP-OH or Hf-TP-SN solution, 900 µL DMSO and 50 µL H_3PO_4 were mixed and sonicated for 1 hour and let stand overnight, after which the mixture was diluted to a proper concentration for UV-Vis measurement. The absorbance of H_2TP-OH at 304 nm was used to calculate the concentration of H_2TP-OH in Hf-TP-OH or Hf-TP-SN after comparison with the standard curve of H_2TP-OH in DMSO, while the absorbance of SN38 at 390 nm was used to calculate the concentration of SN38 in Hf-TP-SN after comparison with the standard curve of SN38 in DMSO.

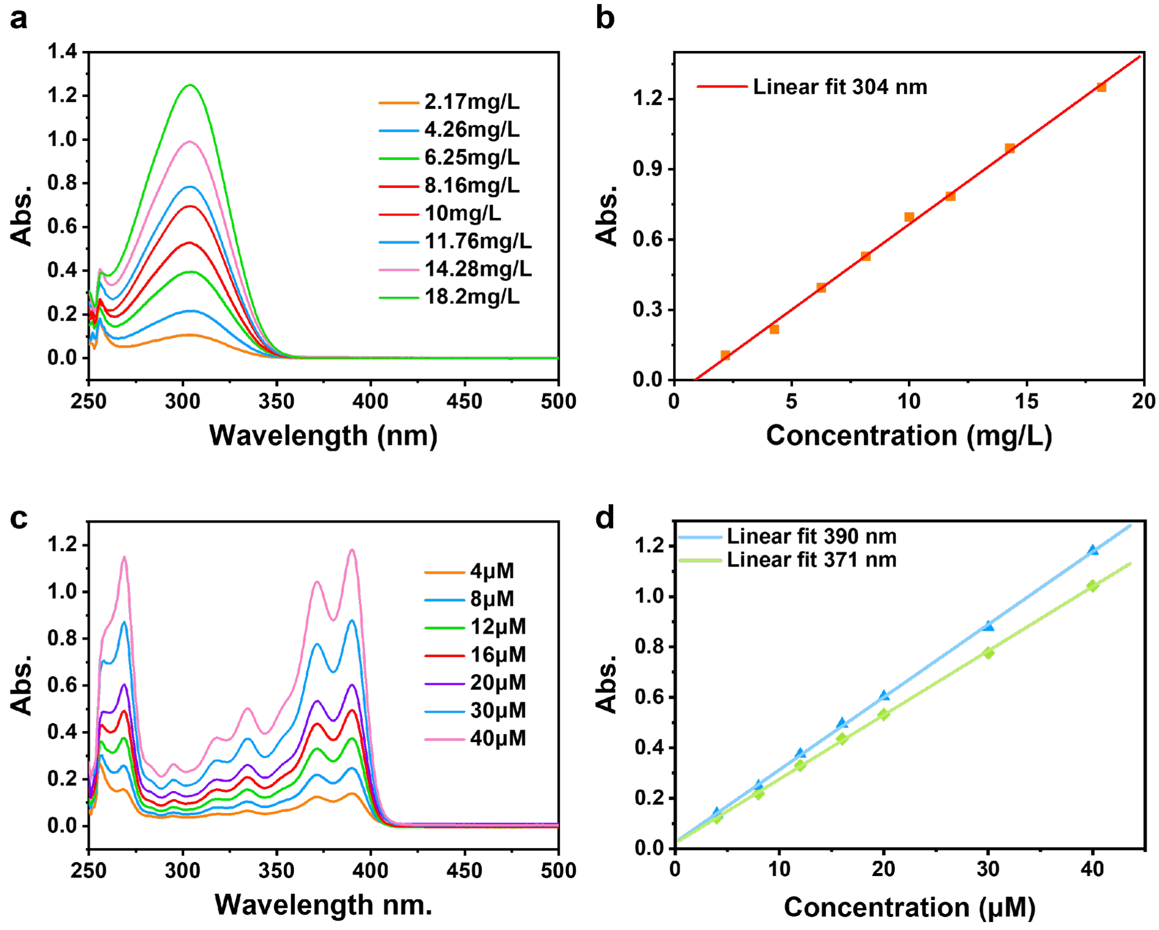

Figure S2. UV-Vis absorption spectra of (a) H_2TP-OH and (c) SN38 and the fitted standard curves of (b) H_2TP-OH and (d) SN38 in DMSO.

S4.3 Digestion of Hf-TP-SN for quantification of SN38 by LC-MS

100 μL 1M $NaHCO_3$ solution was added to 100 μL Hf-TP-SN dispersion in water (total SN concentration: 100 μM). The mixture was sealed and sonicated for 20 minutes, after which 100 μL PBS (200 mM, pH 4.0) was added to adjust the pH to 7. 100 μL saturated NaCl solution and 150 μL ethyl acetate (EA) were then added before vortexing the mixture for 1 minute. The EA layer after centrifugation was analyzed with LC-MS. For HPLC, absorption of SN38 at 380 nm was used to calculate the concentration of free SN38 after comparison with the standard curve of SN38 in EA. For LC-MS, extracted ion chromatogram (EIC) at m/z value of 393.1450 was used to calculate the concentration of free SN38 after comparison with the standard curve of SN38 in EA.

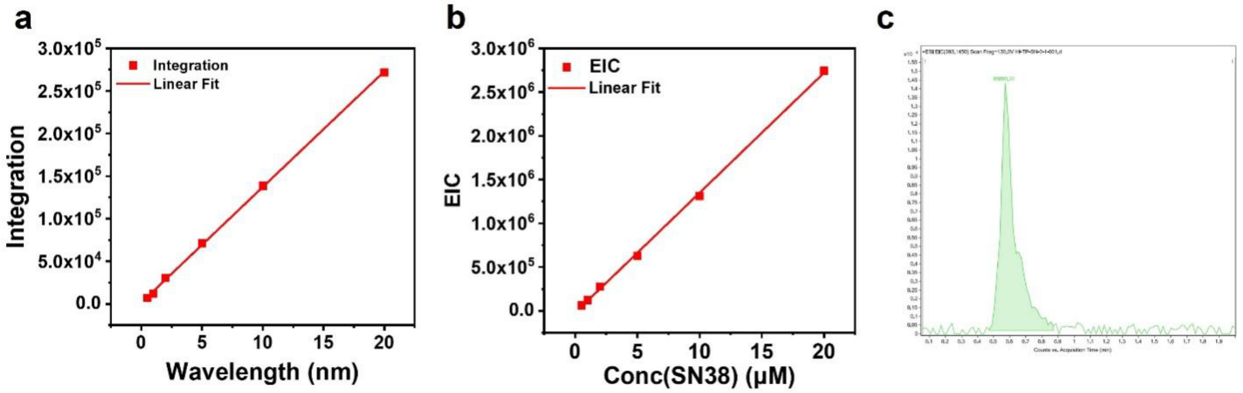

Figure S3. The fitted standard curves of SN38 in EA by (a) HPLC or (b)LC-MS. (c) Representative EIC at m/z value of 393.1450 for quantification of entrapped SN38 in Hf-TP-SN.

S4.4 Stability of Hf-TP-SN in PBS

Hf-TP-SN was dispersed in 1 mL PBS (1 mM) with a Hf concentration of 5.2 mM. 200 uL suspension was taken after incubation for 1, 2, 4, 8, and 24 hours and centrifuged for PXRD measurement.

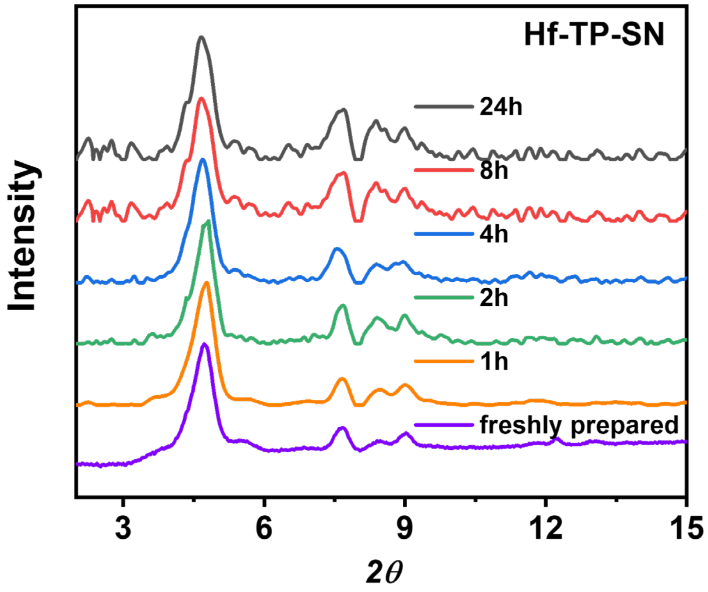

Figure S4. PXRD patterns of Hf-TP-SN freshly prepared and dispersed in PBS for 1, 2, 4, 8 and 24 hours.

S4.5 Stability of Hf-TP-SN after irradiation and long-term storage

Hf-TP-SN was dispersed in 1 mL water with a Hf concentration of 2 mM and then irradiated with 10 Gy X-ray. The morphology, crystallinity, and number-averaged size of the irradiated Hf-TP-SN were examined using TEM, PXRD and DLS, respectively. The Hf-TP-SN nMOF retained the crystallinity and morphology after irradiation as indicated by the same nanoplate morphology in TEM, consistent PXRD pattern, and similar number-averaged sizes. The number-averaged sizes of Hf-TP-SN before and after 10 Gy irradiation were 164 ± 6 nm and 156 ± 5 nm, respectively.

Hf-TP-SN was stored in EA with a Hf concentration of 2 mM for 1 year. The morphology, crystallinity, number-averaged size, and ζ-potential of Hf-TP-SN after long-term storage were examined using TEM, PXRD and DLS. Hf-TP-SN retained the nanoplate morphology under TEM and good crystallinity evidenced by PXRD. The number-averaged size of Hf-TP-SN was 133 ± 8nm, while its ζ -potential was -15.7 ± 0.4 mV, consistent with those of as-synthesized Hf-TP-SN.

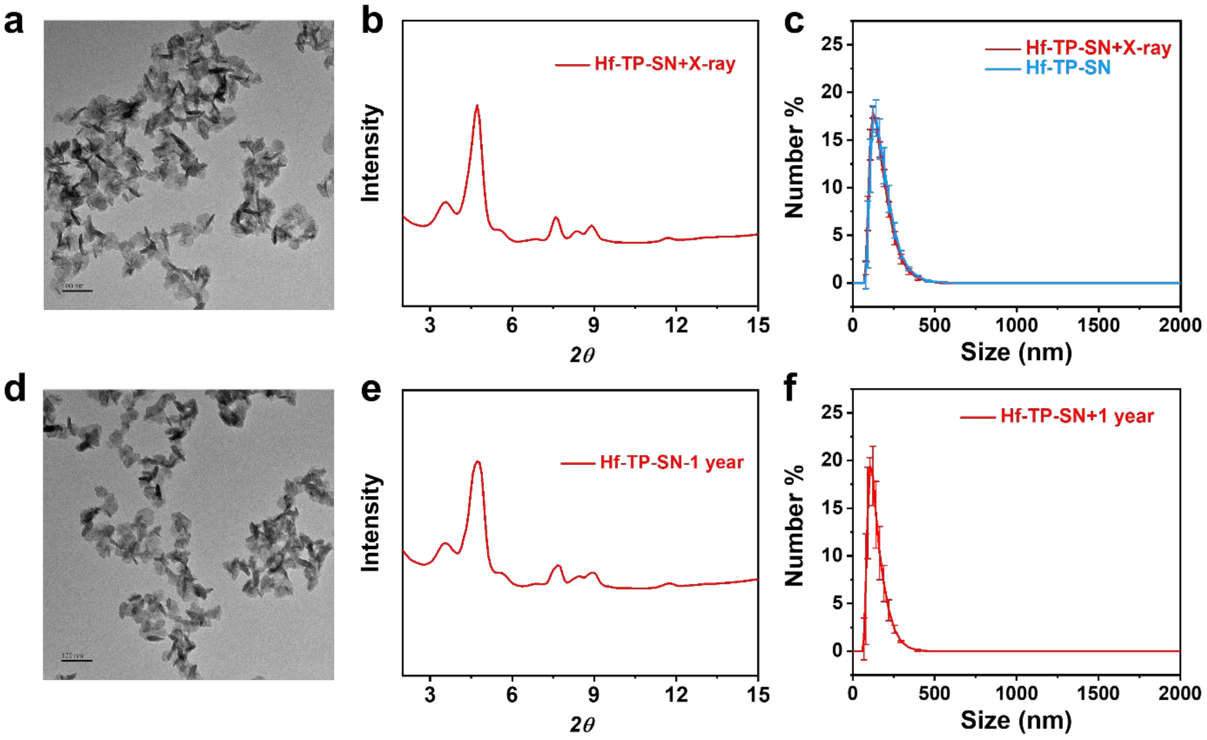

Figure S5. (a) TEM image, (b) PXRD pattern, and (c) number-averaged size of Hf-TP-SN after 10 Gy irradiation (denoted as Hf-TP-SN+X-ray). Number-averaged size of as-synthesized Hf-TP-SN (without X-ray irradiation) is also presented in (c). (d) TEM, (e) PXRD, and (f) number-averaged size of Hf-TP-SN after storing in EA for 1 year.

S4.6 ROS generation in test tubes

Total ROS generation under irradiation was detected by the 2',7'-dichlorodihydrofluorescein (DCFH) assay following reported procedures.[2] 1 mL DCFH-DA (1mM) in DMSO was hydrolyzed by 4 mL NaOH (0.01 M) solution in the dark for 30 minutes and stopped by adding 20 mL PBS (25 mM, pH 7.4). The freshly-prepared DCFH was then added to the PBS suspension (pH 7, 10 mM) of Hf-TP-OH or Hf-TP-

SN at the same Hf concentration. In the final mixture, the concentration of DCFH was 10 μM while the concentration of Hf was 40 μM. The PBS solution with the same DCFH concentration served as a blank control. 100 μL of each suspension was added to 96-well plates (n = 6) and then irradiated with X-ray at 0, 1, 2, 3, 5, or 10 Gy, respectively. The fluorescence signal (em. 520/20 nm) was collected with a Synergy HTX microplate reader (ex. 485/20 nm).

Hydroxyl radical (·OH) generation under irradiation was detected by the APF assay. APF assay was added to the PBS suspension of Hf-TP-OH or Hf-TP-SN at the same Hf concentration. In the final mixture, the concentration of APF was 5 μM while that of Hf was 40 μM. The PBS solution with the same APF concentration served as a blank control. 100 μL of each suspension was added to 96-well plates (n = 6) and then irradiated with X-ray at 0, 1, 2, 3, 5, or 10 Gy, respectively. The fluorescence signal (em. 520/20 nm) was collected with a Synergy HTX microplate reader (ex. 485/20 nm).

S4.7 Hydroxyl radical triggered SN38 release in test tubes

Hf-TP-SN or MeO-SN was dispersed in H_2O at the same concentration of total SN38 (100 μM). $FeCl_3$, (+)-Sodium L-ascorbate, $Na_2(EDTA)\cdot 2H_2O$ (ethylenediaminetetraacetic acid, disodium salt dihydrate) was firstly dissolved in water to reach a concentration of 10 mM separately (10.5 mM for $Na_2(EDTA)\cdot 2H_2O$). Then, 100 μL of each solution and 50 μL H_2O_2 (200 mM in H_2O) was added to Hf-TP-SN or MeO-SN solution to generate hydroxyl radical in situ and to trigger the release of SN38. For control group, only H_2O_2 was added. After 8h incubation at RT, additional 100 μL 1M $NaHCO_3$ was added to Hf-TP-SN to digest the nMOF, after which the mixture was sealed and sonicated for 20 minutes before the addition of 100 μL PBS (200 mM, pH 4) to adjust the pH to 7. 100 μL saturated NaCl solution and 150 μL ethyl acetate (EA) were added before vortexing the Hf-TP-SN or MeO-SN mixture for 1 minute. The EA layer after centrifugation was analyzed by LC-MS.

S4.8 X-ray triggered release in test tubes

Hf-TP-SN or MeO-SN were dispersed in H_2O at the same concentration of total SN38 (100 μM). 100 μL Hf-TP-SN or MeO-SN suspension was irradiated with 10 Gy X-ray. 100 μL 1M $NaHCO_3$ was added to Hf-TP-SN to digest the nMOF, after which the mixture was sealed, sonicated for 20 minutes, and sat 15h before the addition of 100 μL PBS (200 mM, pH 4.0) to adjust the pH to 7. 100 μL saturated NaCl solution and 150 μL ethyl acetate (EA) were added before vortexing the Hf-TP-SN or MeO-SN mixture for 1 minute. The EA layer after centrifugation was analyzed by HPLC.

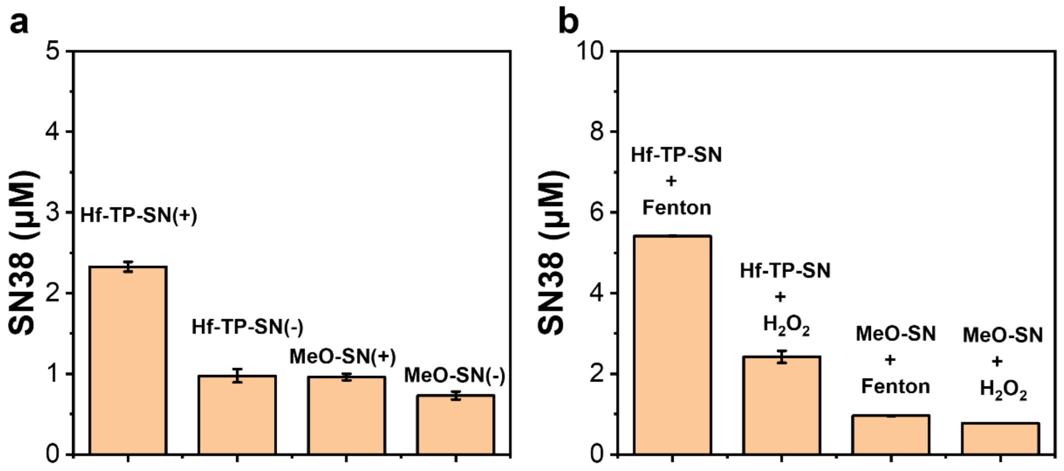

Figure S6. Concentration of SN38 released from MeO-SN or Hf-TP-SN (a) after 10 Gy of X-ray irradiation or (b) after reacting with hydroxyl radical generated by Fenton reaction. Total concentration of SN38 in MeO-SN and Hf-TP-SN was 100 μM. (+) represents 10 Gy X-ray irradiation while (-) represents no irradiation in a. Entrapped SN38 was subtracted.

S4.9 Time-dependent release of SN38 from Hf-TP-SN with or without X-ray irradiation

Hf-TP-SN were dispersed in PBS (0.1 ×, pH = 7.4) at a total SN38 concentration of 100 μM. Half of Hf-TP-SN suspension was irradiated with 10 Gy X-ray. At different timepoints, 100 μL saturated NaCl solution and 150 μL ethyl acetate were added to a 100 μL Hf-TP-SN dispersion stored at 4 °C (with or without irradiation) before vortexing the mixture for 1 minute. The EA layer after centrifugation was analyzed with LC-MS. Hf-TP-SN without irradiation slowly released the entrapped SN38 (~2.9% after 24 h; the entrapped SN38 for this sample was determined to be 2.6%), while Hf-TP-SN after 10 Gy irradiation released more SN38 (by 0.70% ~ 1.56%) due to X-ray triggered release of SN38.

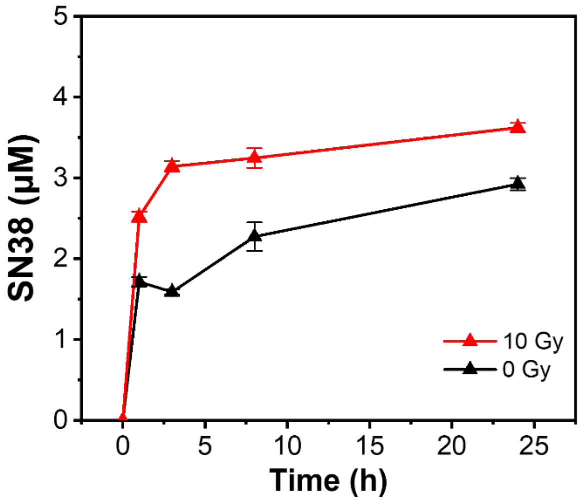

Figure S7. Time-dependent release of SN38 from Hf-TP-SN with or without 10 Gy X-ray irradiation. Starting Hf-TP-SN concentration was 100 μM, n=4.

S5. In Vitro Experiments

S5.1. Dark toxicity

CT26 cells were cultured in RPMI-1640 medium supplemented with 10% fetal bovine serum, 1% HyClone penicillin-streptomycin 100X solution, and cultured in a humidified atmosphere containing 5% CO_2 at 37°C. 4T1 cells were cultured in RPMI-1640 medium supplemented with 10% fetal bovine serum and 1% HyClone penicillin-streptomycin 100X solution. MC38 cells were cultured in DMEM medium supplemented with 10% fetal bovine serum, 1% HyClone penicillin-streptomycin 100X solution, 1% sodium pyruvate solution, 1% Hepes solution, and 1% Pen/Strep 100X solution. The cytotoxicity of H_2TP-OH, Hf-TP-OH, and Hf-TP-SN on CT26 cells was detected by MTS assay. CT26 cells were seeded in 96-well plates at a density of 2500 cells/well. Different concentrations of H_2TP-OH, Hf-TP-OH and Hf-TP-SN were added and 24 hours later 10% (V/V) of MTS reagent was added to each well. 90 minutes later the absorbance of each well at 490 nm was read by a Synergy HTX plate reader to calculate cell viability.

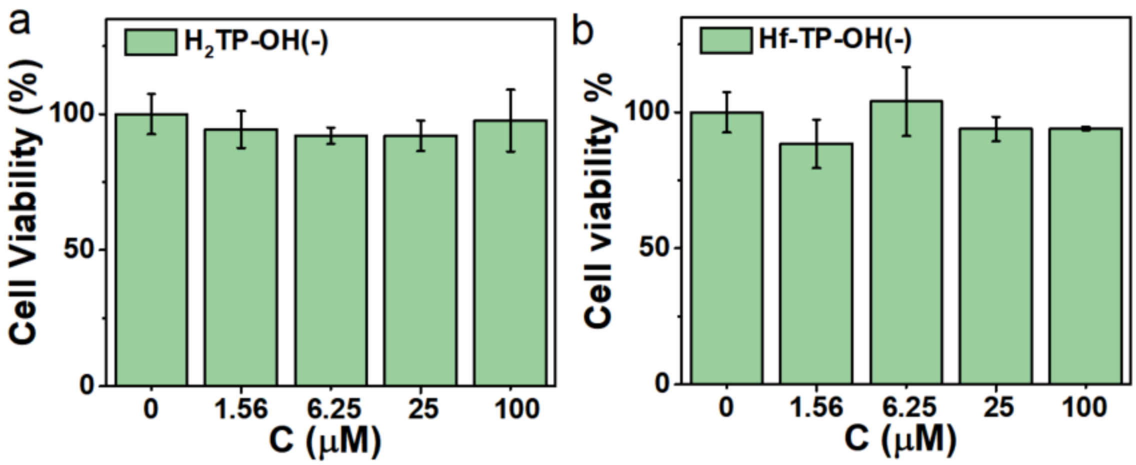

Figure S8. Cell viability of CT26 cells treated by (a) H_2TP-OH(-)or (b) Hf-TP-OH(-).

S5.2. Clonogenic assay

CT26 cells were seeded in 6-well plates at a density of 1.5 × 10^5 cells/well and cultured overnight. The cells were incubated with PBS, Hf-TP-OH, or Hf-TP-SN at an equivalent metal concentration of 50 μM for 4 hours, and then irradiated with 0, 2, 4, 6 and 8 Gy X-ray (n = 3). The cells were washed with PBS twice and then trypsinized to afford single cell suspensions. The cells were counted and diluted, then 200 cells were seeded in each well of 6-well plates and cultured in 2 mL medium for another 7 days. Then, the plates were rinsed once with PBS, fixed by 4% paraformaldehyde for 20 minutes, and washed with PBS twice at RT. After that, the 6-well plates were scanned with IncuCyte S3 in the whole well mode with a 4× objective. Clonogenic assay of 4T1 and MC38 cells was also conducted according to the same method as mentioned above. The colonies were identified with IncuCyte 2021A software in a cellular resolution and the confluence was used as a parameter to calculate the plating efficiency (PE) and surviving fraction (SF):

PE = Confluence (*0 Gy, PBS*)/Cell # (*0 Gy, PBS*)

SF (D, MOF) = Confluence (*D, MOF*)/Cell # (*D, MOF*)×PE

Where D was the radiation dose and Cell # (*D, MOF*) was the number of cells seeded for a certain radiation dose D and a certain treatment group.

The dose modifying ratio at a 10% ($DMR_{10\%}$) was used as a parameter to assess radiosensitization effect and defined as the ratio of doses under reference conditions to produce a 10% :

$$DMR_{10\%} = DMR_{10\%}\ (PBS)\ /\ DMR_{10\%}\ (MOF)$$[3]

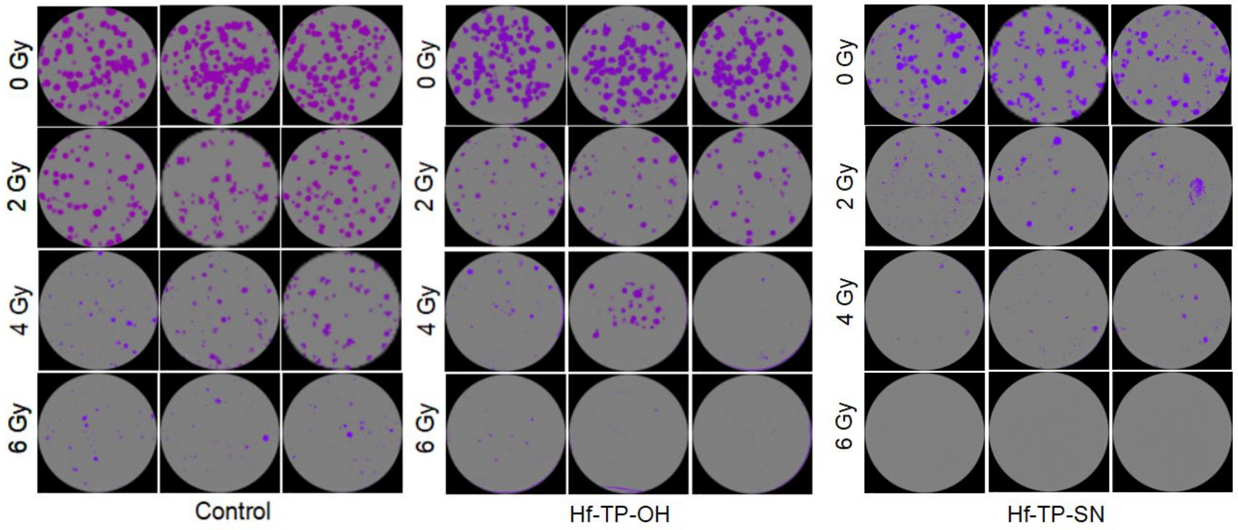

Figure S9. Representative images of colonies formed by X-ray treated CT26 cells pre-incubated with PBS, Hf-TP-OH or Hf-TP-SN (well diameter = 35 mm).

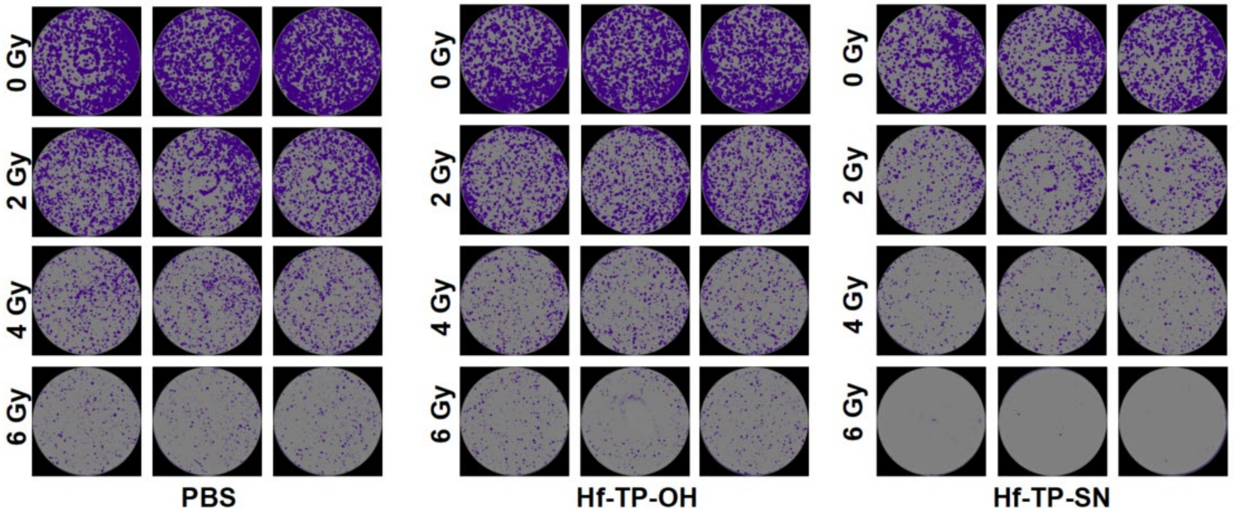

Figure S10. Representative images of colonies formed by X-ray treated 4T1 cells pre-incubated with PBS, Hf-TP-OH, or Hf-TP-SN (well diameter = 35 mm).

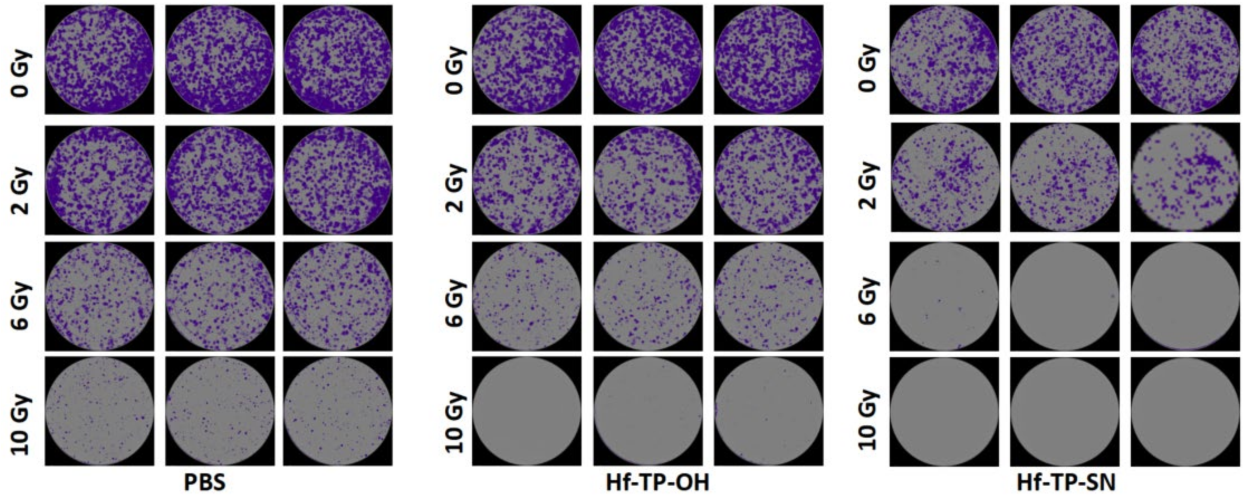

Figure S11. Representative images of colonies formed by X-ray treated MC38 cells pre-incubated with PBS, Hf-TP-OH, or Hf-TP-SN (well diameter = 35 mm).

S5.3. Hydroxyl radical generation

To demonstrate ·OH generation, CT26 cells were seeded in cell culture dishes at a density of 1.5×10^5 and cultured overnight. Hf-TP-OH was added at an equivalent Hf concentration of 50 μM and further incubated in a 37℃ incubator for 4 hours. The cells were washed with PBS solution 3 times and 1 mL cell culture medium containing with 10 μM HPF was added and incubated at 37℃ for another 30 minutes. Then the cells were irradiated with X-ray (3 Gy) and cultured in the cell incubator for another 24 h. After that, the cells were washed with PBS for three times and further incubated with Hoechst 33342 (10 μg mL^{-1}) in PBS for 10 minutes in the cell incubator. Finally, the cells were washed with PBS 3 times and observed on a Leica Stellaris 8 confocal microscope.

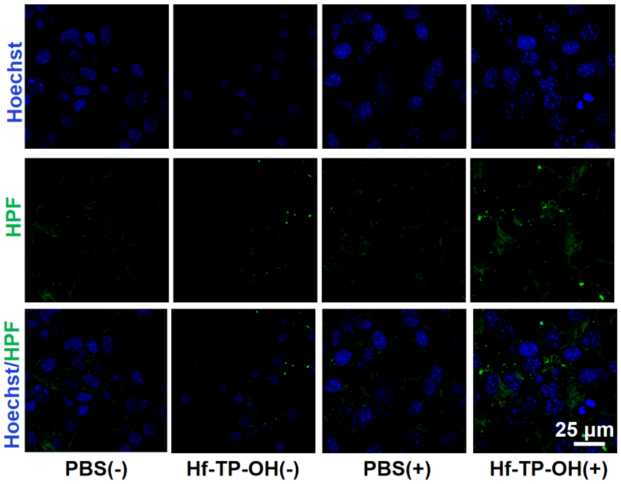

Figure S12. CLSM images of CT26 cells stained by hydroxyphenyl fluorescein (HPF, green) and Hoechst 33342 (blue, cell nucleus) for detecting the generation of ·OH.

S5.4. Dark toxicity

The cytotoxicities of SN38 and Me_2TP-SN on CT26 cells were detected by MTS assay. CT26 cells were seeded in 96-well plates at a density of 2500 cells/well. Different concentrations of SN38 and Me_2TP-SN were added and 48 hours later, 10% (v/v) of MTS reagent was added to each well. 90 minutes later the absorbance of each well at 490 nm was read by a Synergy HTX plate reader to calculate cell viability.

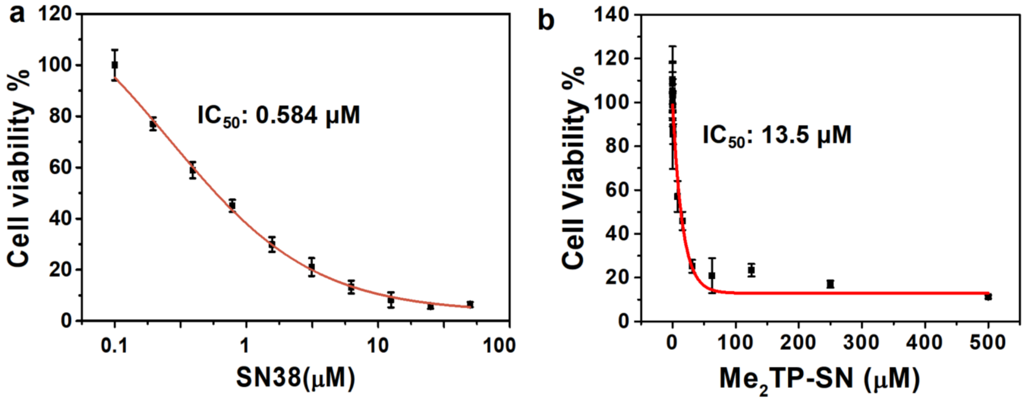

Figure S13. Cell viability curves of CT26 cells treated by (a) SN38(-)or (b) Me_2TP-SN38(-).

S5.5. Cellular uptake

The cellular uptake of Hf-TP-SN (Hf: 50 μM) was evaluated on CT26 cells. The cells were seeded in 6-well plates at a density of 2 × 10^5/well and cultured overnight. Hf-TP-SN was added at a Hf concentration of 50 μM into the medium (n = 3). The cells were incubated in a 37 ℃ incubator for 1, 2, and 6 hours. At each time point, the medium was aspirated, the cells were washed with PBS three times, collected by centrifugation, and counted with a hemocytometer. The cell pellets were digested with nitric acid for 24 h and the concentration of Hf was detected by ICP-MS.

To probe the endocytosis mechanism, cells were seeded in 6-well plates at a density of 2 × 10^5/well and cultured overnight. 100 μM chlorpromazine, 270 μM nystatin, or 5 μM rottlerin was added to CT26 cells and incubated for 1 h to inhibit clathrin, caveolae, or macropinocytosis-mediated endocytotic pathway, respectively. Then, Hf-TP-SN was added at a Hf concentration of 25 μM into the medium (n = 3). The cells were incubated in a 37 ℃ incubator for 24 hours. Cell pellets were digested with nitric acid for 24 h and the concentration of Hf was detected by ICP-MS.

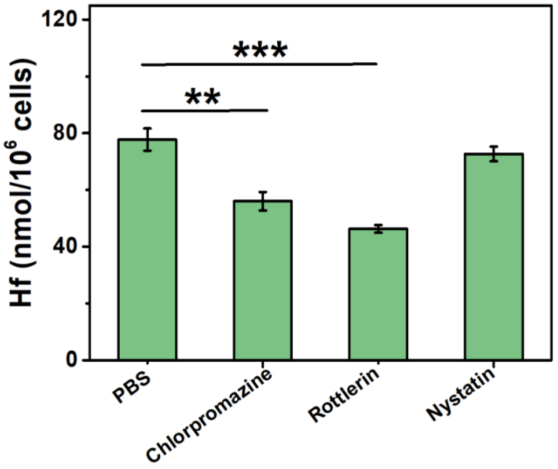

Figure S14. Endocytosis inhibited by chlorpromazine, nystatin, or rottlerin.

The inhibition by chlorpromazine and rottlerin indicates that the uptake of Hf-TP-SN into CT26 cells is through clathrin-dependent pathway and macropinocytosis.

S5.6. ROS generation

To demonstrate the generation of ROS, CT26 cells were seeded in cell culture dishes at a density of 1.5×10^5 and cultured overnight. Hf-TP-SN or Hf-TP-OH was added at a Hf concentration of 50 μM and further incubated in a 37℃ incubator for 4 hours. The cells were washed with PBS solution 3 times and 1 mL cell culture medium containing with 30 μM DCFH-DA was added and incubated at 37℃ for another 30 minutes. Then the cells were irradiated with X-ray (3 Gy) and cultured in the cell incubator for another 24 h. After that, the cells were washed with PBS three times and further incubated with Hoechst 33342 (10 μg mL^{-1}) in PBS for 10 minutes. Finally, cells were washed with PBS 3 times and observed on a Leica Stellaris 8 confocal microscope. The DCF^+ cells were also quantified by flow cytometry (FITC channel).

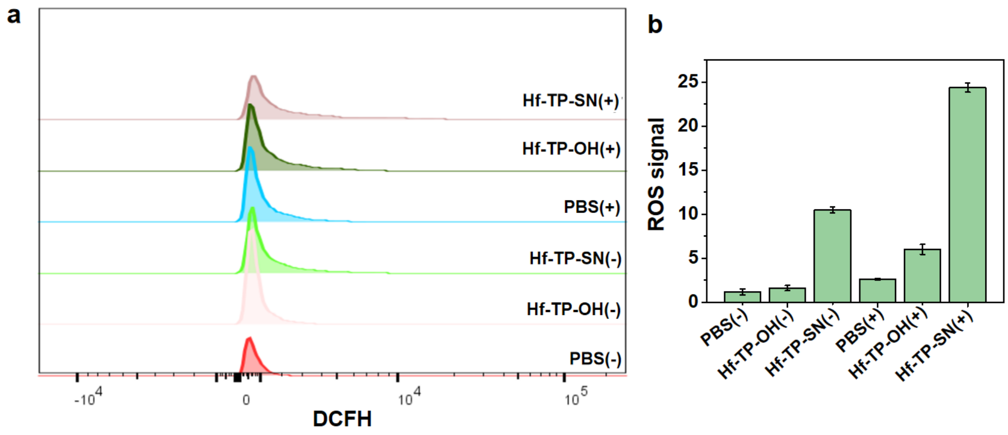

Figure S15. (a) Quantification of histograms of intracellular ROS signals by flow cytometry. (b) Percentage of DCF^+ CT26 cells after Hf-TP-OH(+) or Hf-TP-SN(+) treatment. X-ray dose was 3 Gy.

Table S1. Percentage of DCF^{+} CT26 cells after different treatments. X-ray dose was 3 Gy (Figure S15b).

Treatment	DCF^{+} %
PBS(-)	1.18
Hf-TP-OH(-)	1.62
Hf-TP-SN(-)	10.5
PBS(+)	2.62
Hf-TP-OH(+)	6.00
Hf-TP-SN(+)	24.37

S5.7. DNA damage

For CLSM imaging, CT26 cells were seeded in cell culture dishes at a density of 1.5×10^{5}. The cells were treated in the same way as in the clonogenic assay. 24 hours after radiation, the cells were washed with PBS and fixed with 4% paraformaldehyde at RT for 20 minutes. The cells were again rinsed with PBS, blocked and permeabilized with 5% FBS + 0.3% Triton-X in PBS at RT for 1 hour. After blocking, cells were incubated with the γ-H2AX primary antibody (1:500) in 1% BSA + 0.3% Triton-X in PBS at RT for 1 hour. The cells were then washed with PBS and incubated with the Alexa Fluor 488 conjugated secondary antibody (1:3000) in 1% BSA + 0.3% Triton-X in PBS at RT for 1 hour. Afterwards, the cells were washed with PBS and further incubated with Hoechst 33342 (10 μg mL^{-1}) in PBS for 10 minutes at 37 ^{o}C to visualize cell nuclei, respectively. Finally, cells were washed with PBS 3 times and observed on a Leica Stellaris 8 confocal microscope.

S5.8. Apoptotic cell death

To quantify apoptosis, CT26 cells were seeded in 6-well plates at a density of 1.5×10^{5}/well and cultured overnight. Hf-TP-OH or Hf-TP-SN was added at an equivalent metal concentration of 50 μM for 4 hours and irradiated with X-ray (3 Gy). 24 hours later, the cells were washed with PBS, trypsinized to afford single cell suspensions, cells were stained with the dead cell apoptosis kit with annexin V Alexa Fluor 488 & PI following the manufacturer's protocol and resuspended in the binding buffer for flow cytometric analysis (Annexin-V in FITC channel, PI in PE-dazzle 594 channel).

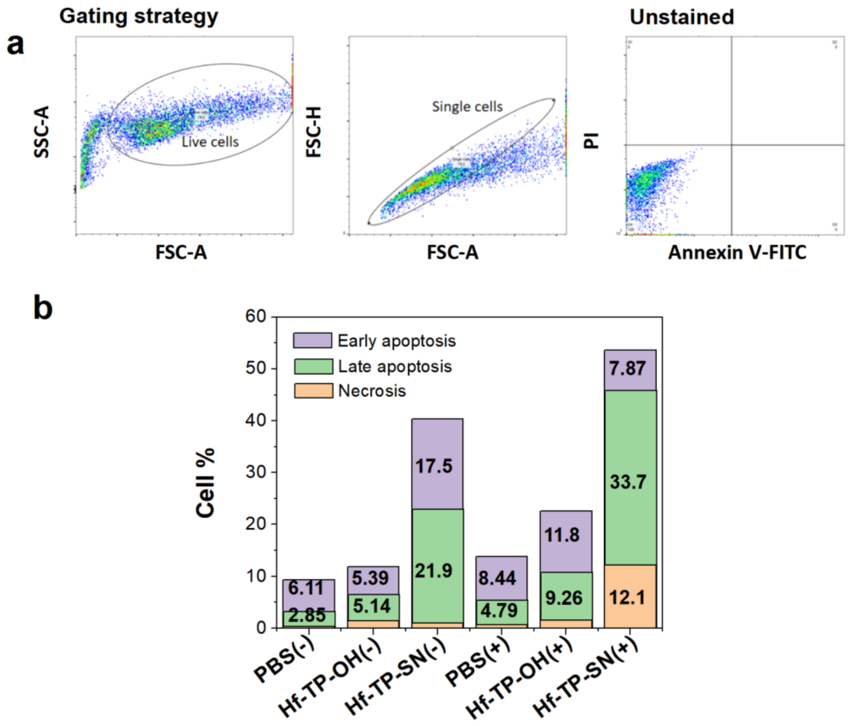

Figure S16. (a) Gating strategy for the analysis of apoptotic cell death in PBS(-) group without staining. (b) Percentage of early apoptotic, late apoptotic, and necrotic CT26 cells after different treatments. X-ray dose was 3 Gy.

Owing to the enhanced RT effect and successful release of SN38, Hf-TP-SN(+) upregulated phosphatidylserine on cell membranes by Annexin-V staining, and impaired cell membrane function with influx of cell-impermeable propidium iodide (PI) via the synergistic chemotherapy and radiotherapy. Hf-TP-SN(+) gave 3.64-and 7.04-fold higher late apoptotic cell death than Hf-TP-OH(+) or PBS(+) treatment, respectively.

S5.9. In vitro drug release

The release of SN38 from Hf-TP-SN on CT26 cells was detected by MTS assay. CT26 cells were seeded in 96-well plates at a density of 2500 cells/well. Different concentrations of Hf-TP-SN were dispersed in cell culture medium and irradiated by X-ray with different doses (6 and 60 Gy). After that, 100 mL of above Hf-TP-SN containing culture medium was added to CT26 cells. 48 hours later, 10% (V/V) of MTS reagent was added to each well. 90 minutes later the absorbance of each well at 490 nm was read by a Synergy HTX plate reader to calculate cell viability.

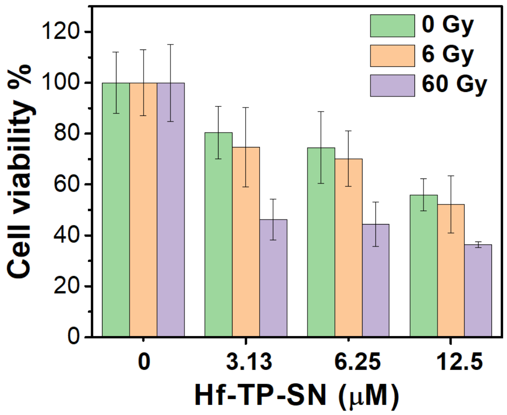

Figure S17. Cell viability of CT26 cells treated by Hf-TP-SN pre-irradiated with different doses of X-ray.

S6. In Vivo Experiments

S6.1. Anti-cancer efficacy

BALB/c mice (6-8 weeks) were obtained from Charles River Laboratories, Inc (USA) and bred in house at the animal facility at the University of Chicago. The study protocol was reviewed and approved by the Institutional Animal Care and Use Committee (IACUC) at the University of Chicago. To evaluate the in vivo therapeutic efficacy, CT26 tumor models were established on BALB/c mice by inoculating 2 × 10^6 cells/mouse subcutaneously onto the right flanks at day 0, respectively. When CT26 tumors reached ~85 mm^3, the mice were randomized for treatments. PBS, irinotecan, Hf-TP-OH, or Hf-TP-SN was intratumorally injected with an equivalent metal dose of 0.5 μmol in 20 μL PBS and SN38 dose of 0.047 μmol in 20 μL PBS. The dose of irinotecan was 0.047 μmol in 20 μL PBS. 6-8 hours later, the mice were anaesthetized with 2.5% (v/v) isoflurane/O_2 and mounted onto the X-Rad 225 irradiator. The CT26 tumors were irradiated with 2 Gy X-ray/fraction for 3 consecutive days.

4T1 tumor models were established on BALB/c mice by inoculating 2 × 10^6 cells/mouse subcutaneously onto the right flanks at day 0, respectively. When 4T1 tumors reached ~100-120 mm^3, the mice were randomized for treatments. PBS, Hf-TP-OH, or Hf-TP-SN was intratumorally injected with an equivalent metal dose of 0.5 μmol in 20 μL PBS and SN38 dose of 0.047 μmol in 20 μL PBS. The dose of irinotecan was 0.047 μmol in 20 μL PBS. 6-8 hours later, the mice were anaesthetized with 2.5% (v/v) isoflurane/O_2 and mounted onto the X-Rad 225 irradiator. The 4T1 tumors were irradiated with 2 Gy X-ray/fraction for 3 consecutive days.

The length and width of tumor tissues were measured with an electronic caliper and body weights were monitored with an electronic scale. At the endpoint of the experiments, the mice were euthanized, and the tumors and major organs were sectioned for hematoxylin-eosin (H&E) staining to evaluate general toxicity. The tumor growth inhibition index (TGI) was defined as the equation below:[4]

$$V = L \times W^2 / 2$$

$$TGI = 1 - \frac{m_s}{m_c}$$

Where V, L, W, m_s and m_c represent tumor volume, tumor length, tumor width, average tumor weight of treated mice at endpoint, control mice at endpoint, respectively.

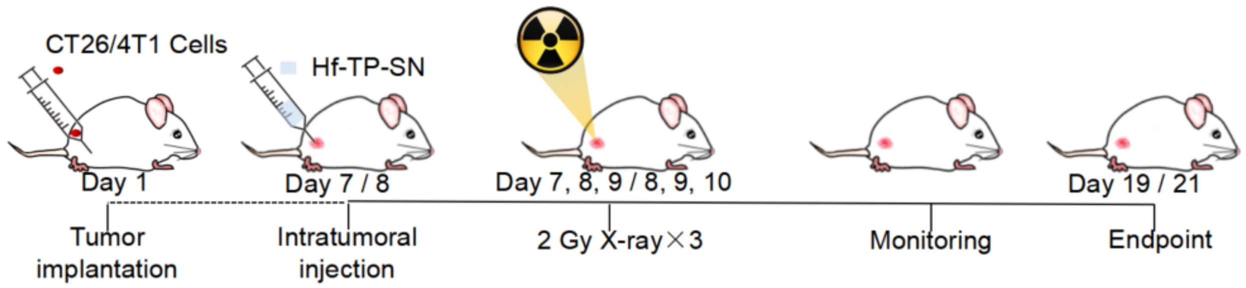

Figure S18. Schematic illustration of the dosing schedule on subcutaneous CT26 and 4T1 tumors in BALB/c mice.

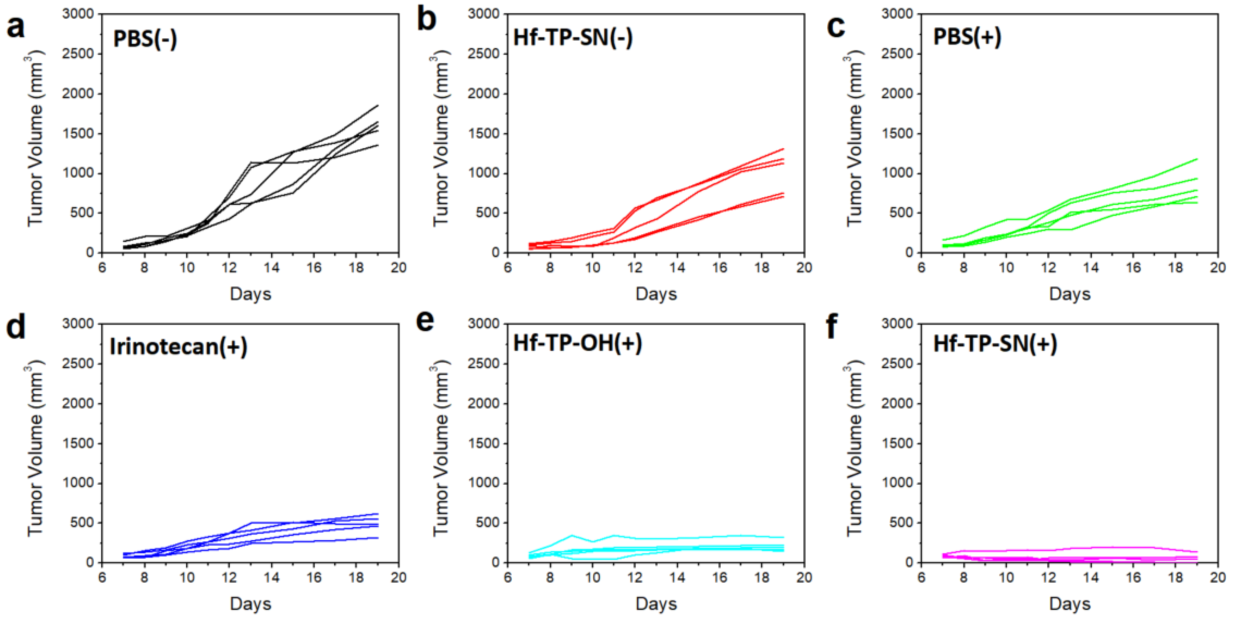

Figure S19. Individual tumor growth curves of CT26-bearing BALB/c mice in **Figure 4a** (n = 5).

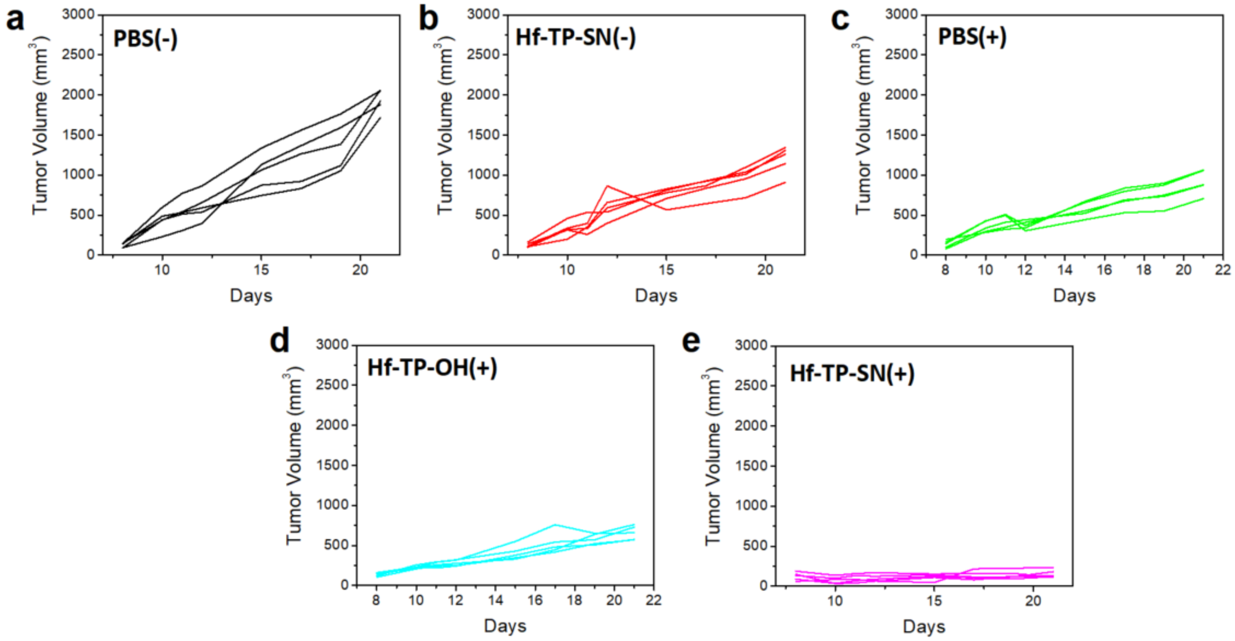

Figure S20. Individual tumor growth curves of 4T1-bearing BALB/c mice in **Figure 4d** (n = 5).

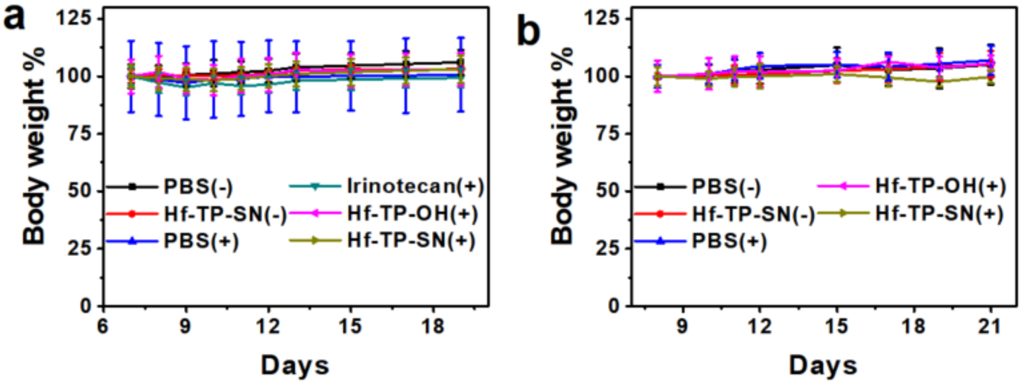

Figure S21. Relative body weights of (a) CT26 and (b) 4T1 tumor-bearing BLAB/c mice after different treatments.

Table S2. TGI values of CT26-bearing BABL/c mice on day 19.

Treatment	TGI (CT26)
Hf-TP-SN(-)	0.362
PBS(+)	0.468
Irinotecan(+)	0.695
Hf-TP-OH(+)	0.869
Hf-TP-SN(+)	0.965

Table S3. TGI values of 4T1-bearing BABL/c mice on day 21.

Treatment	TGI (4T1)
Hf-TP-SN(-)	0.162
PBS(+)	0.328
Hf-TP-OH(+)	0.721
Hf-TP-SN(+)	0.889

S6.2. Immunohistochemistry analysis

To evaluate DNA damage and tumor proliferation after different treatments, a group of CT26-bearing mice were established and treated in the same way as in antitumor efficacy experiments. The mice were euthanized one day after the last irradiation. The tumors were excised and fixed in 4% PFA for 48 h and 70% ethanol for 1 day. The tissues were embedded in paraffin, sectioned and stained for H&E, γ-H2AX, Ki67 and TUNEL by Human Tissue Resource Center at the University of Chicago. Briefly, the slides were deparaffinized and rehydrated using xylenes and serial dilutions of ethanol to distilled water. Then the slides were treated with antigen retrieval buffer (Leica Biosystems, AR9640) and heated in a steamer at 97°C for 20 minutes. After washing with tris-buffered saline (TBS), the slides were incubated with primary γ-H2AX antibody (1:400) or primary Ki67 antibody (Thermo Fisher Scientific, Clone# SP6, 1:400) at RT for 1 hour in a wet chamber. The slides were washed with TBS, then γ-H2AX and Ki67 slides were incubated with anti-rabbit-polymer (Bond Polymer Refine Detection, Leica Biosystems, DS9800) for 30 minutes at RT. The antigen-antibody binding was detected with the 3,3′-Diaminobenzidine (DAB) (DAKO, K3468) system. Tissue sections were then immersed in hematoxylin for counterstaining and covered with cover glasses. The slides were scanned on a CRi Pannoramic SCAN 40× whole slide scanner by Integrated Light Microscopy Core at the University of Chicago. The images were analysed with QuPath-0.2.3 AND Image J software.

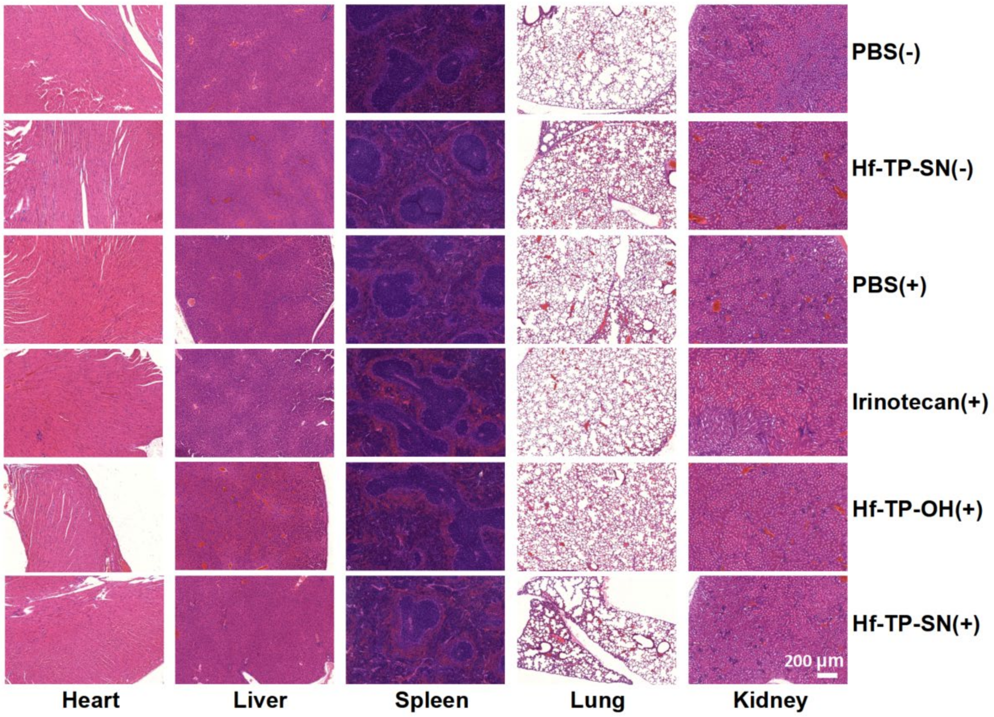

Figure S22. H&E staining of hearts, livers, spleens, lungs and kidneys of CT26 tumor-bearing BALB/c mice in different treatment groups (scale bar: 200 μm).

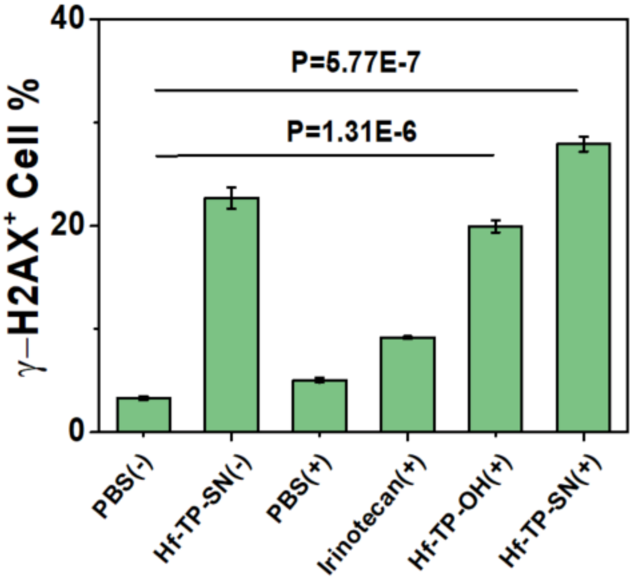

Figure S23. Statistical analysis of positive rates of γ-H2AX staining in CT26 tumor slides.

γ-H2AX staining indicated that Hf-TP-SN(+) caused 1.4-fold more DSBs than Hf-TP-OH(+) due to the successful X-ray triggered release of SN38.

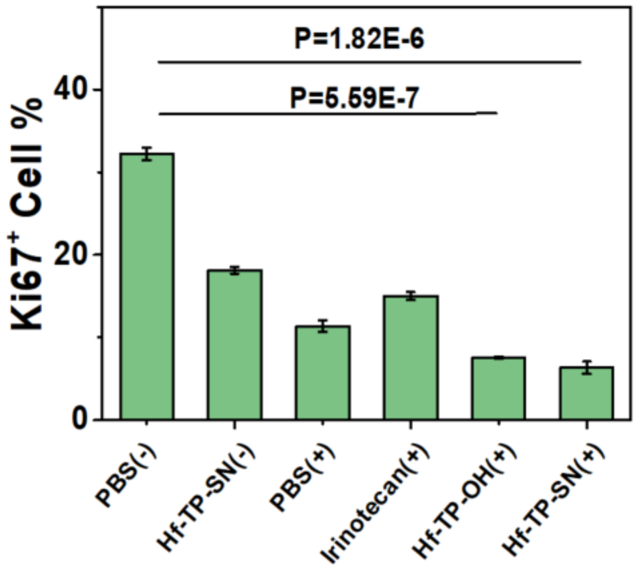

Figure S24. Statistical analysis of positive rates of Ki67 staining in CT26 tumor slides.

Hf-TP-OH(+) induced a 1.99-fold stronger inhibition of tumor proliferation than PBS(+), while Hf-TP-SN(+) induced a 2.37-fold stronger inhibition of tumor proliferation than PBS(+), suggesting the combination of radiotherapy and chemotherapy could enhance the inhibition of tumor proliferation.

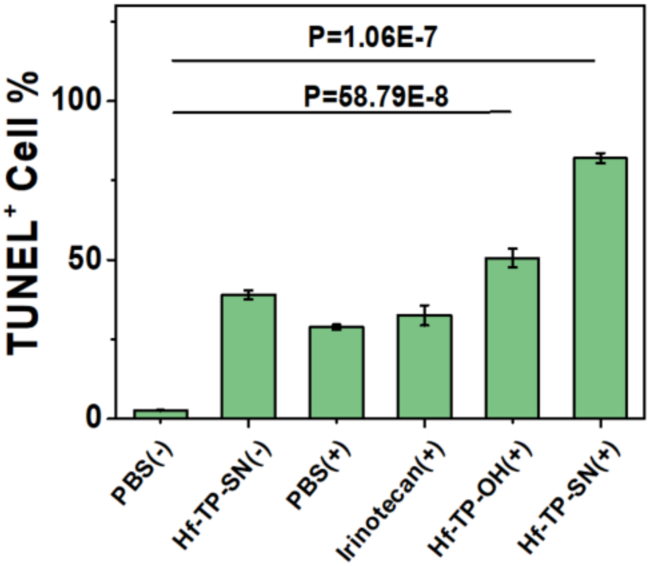

Figure S25. Statistical analysis of positive rates of TUNEL staining in CT26 tumor slides.

Owing to the enhanced RT effect and successful release of SN38, Hf-TP-SN(+) treated tumor showed the highest ratio of $TUNEL^+$ cancer cells due to the synergistic chemotherapy and radiotherapy.

S7. NMR spectra

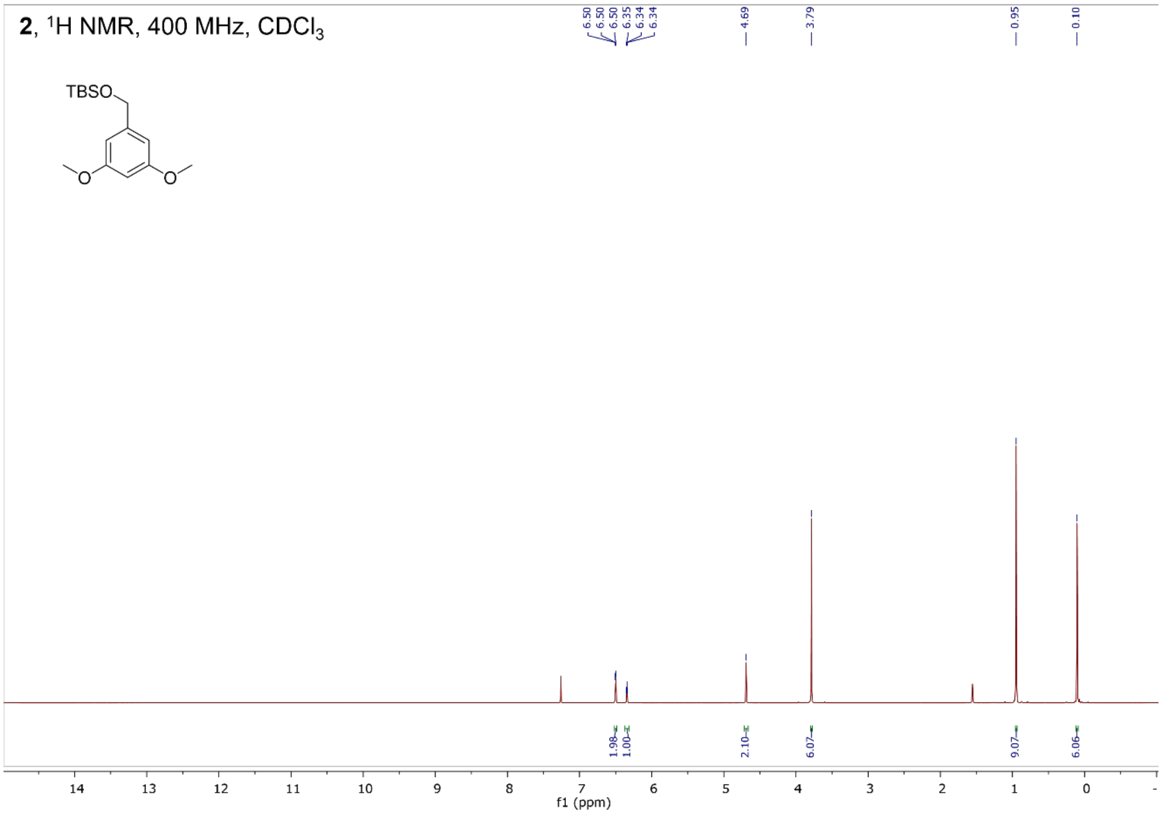

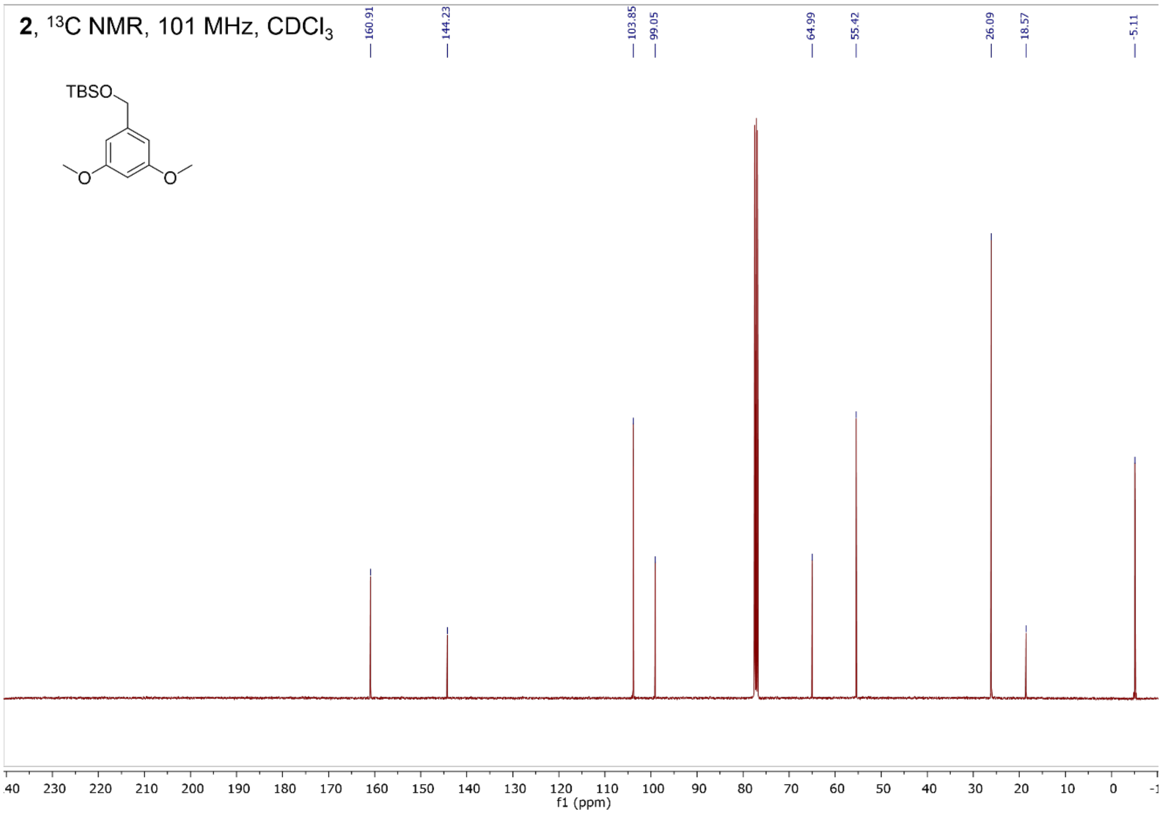

3, ^{1}H NMR, 400 MHz, $CDCl_3$

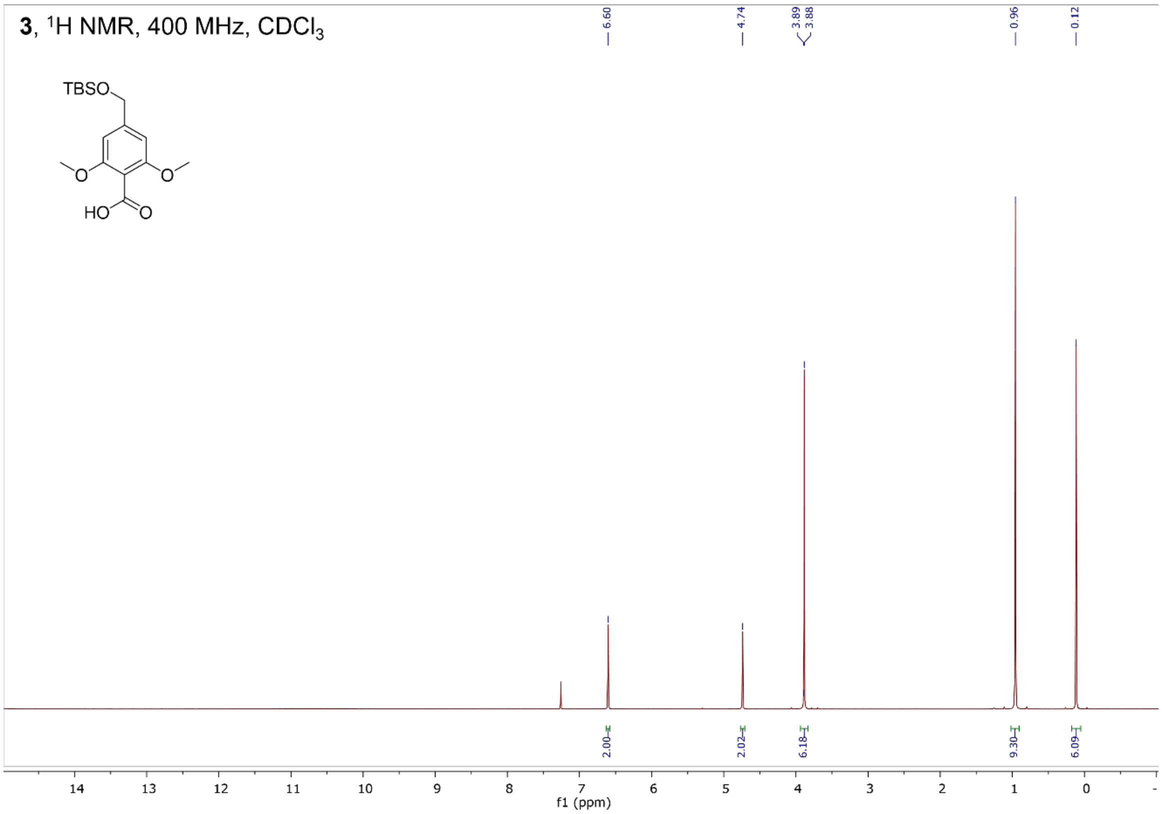

3, ^{13}C NMR, 101 MHz, $CDCl_3$

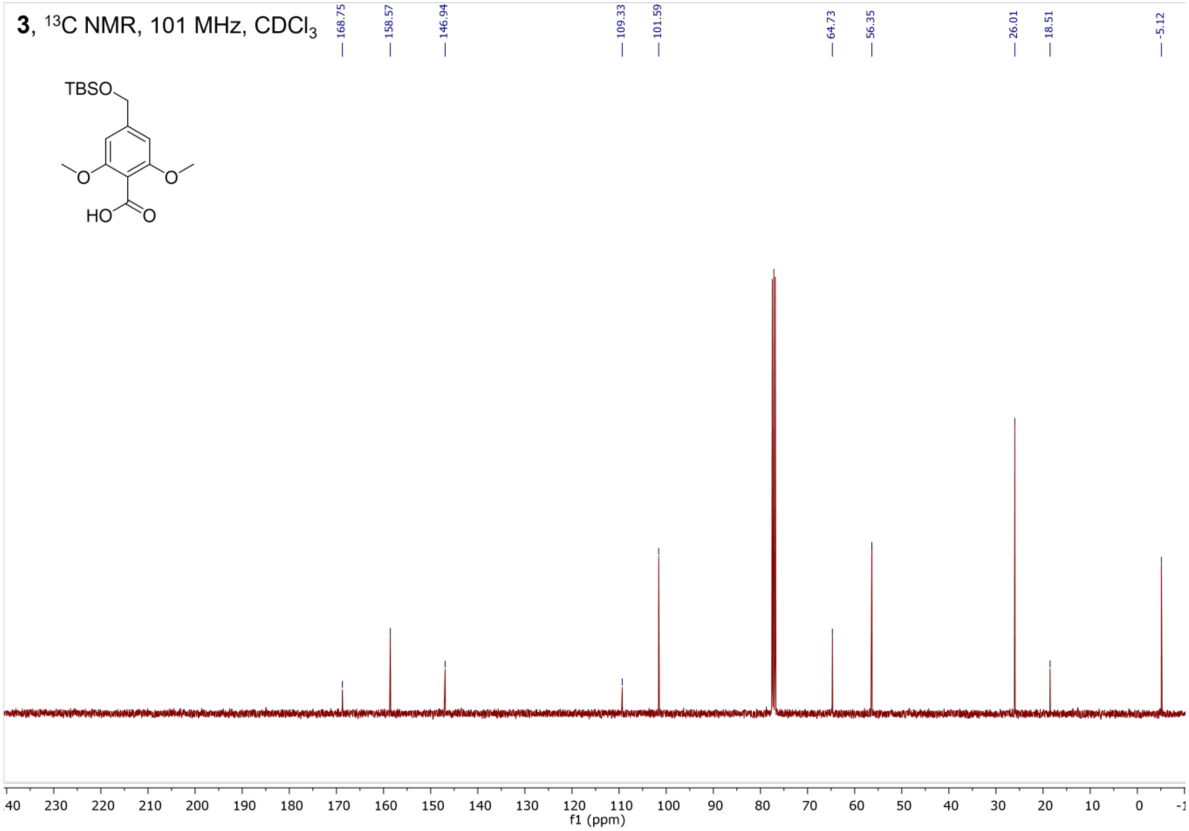

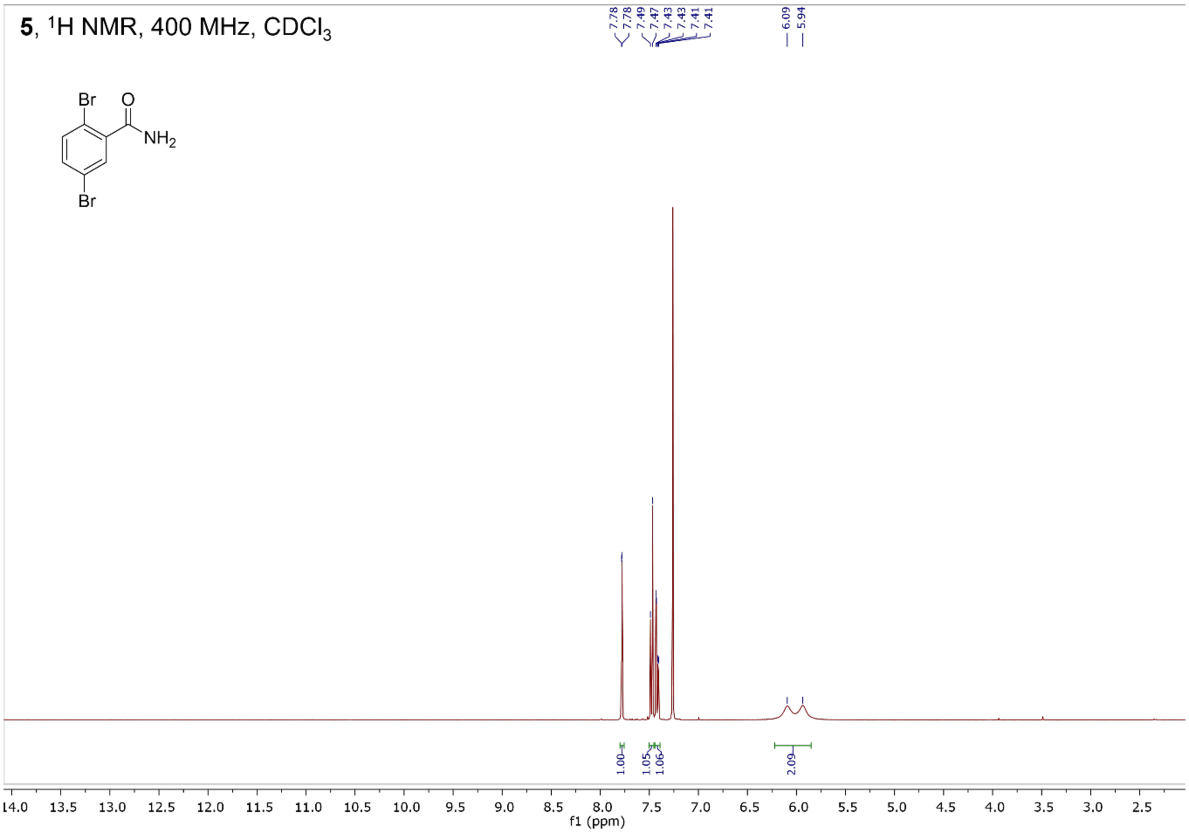
5, 1H NMR, 400 MHz, CDCl3
7.78
7.78
7.49
7.47
7.43
7.43
7.41
7.41
6.09
5.94
Br
O
NH2
Br
1.00
1.05
1.06
2.09
14.0 13.5 13.0 12.5 12.0 11.5 11.0 10.5 10.0 9.5 9.0 8.5 8.0 7.5 7.0 6.5 6.0 5.5 5.0 4.5 4.0 3.5 3.0 2.5
f1 (ppm)

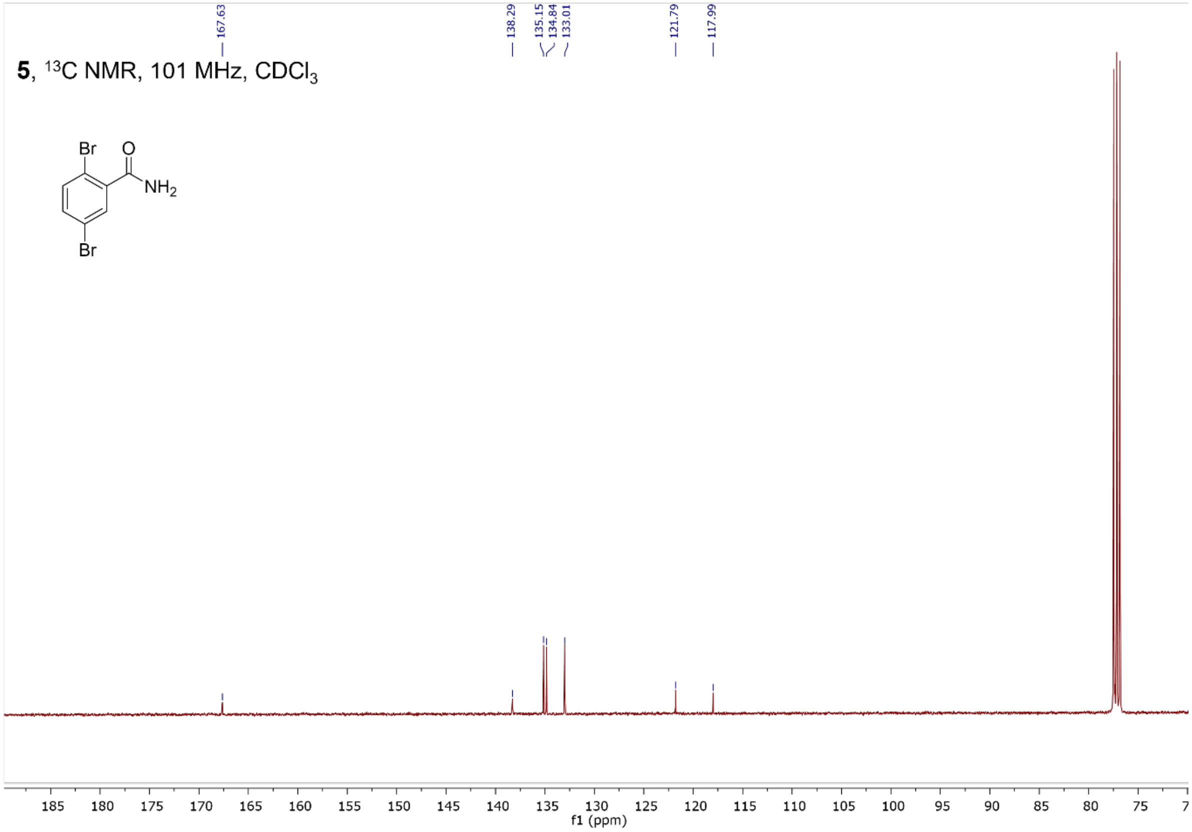
167.63
138.29
135.15
134.84
133.01
121.79
117.99
5, 13C NMR, 101 MHz, CDCl3
Br
O
NH2
Br
185 180 175 170 165 160 155 150 145 140 135 130 125 120 115 110 105 100 95 90 85 80 75
f1 (ppm)

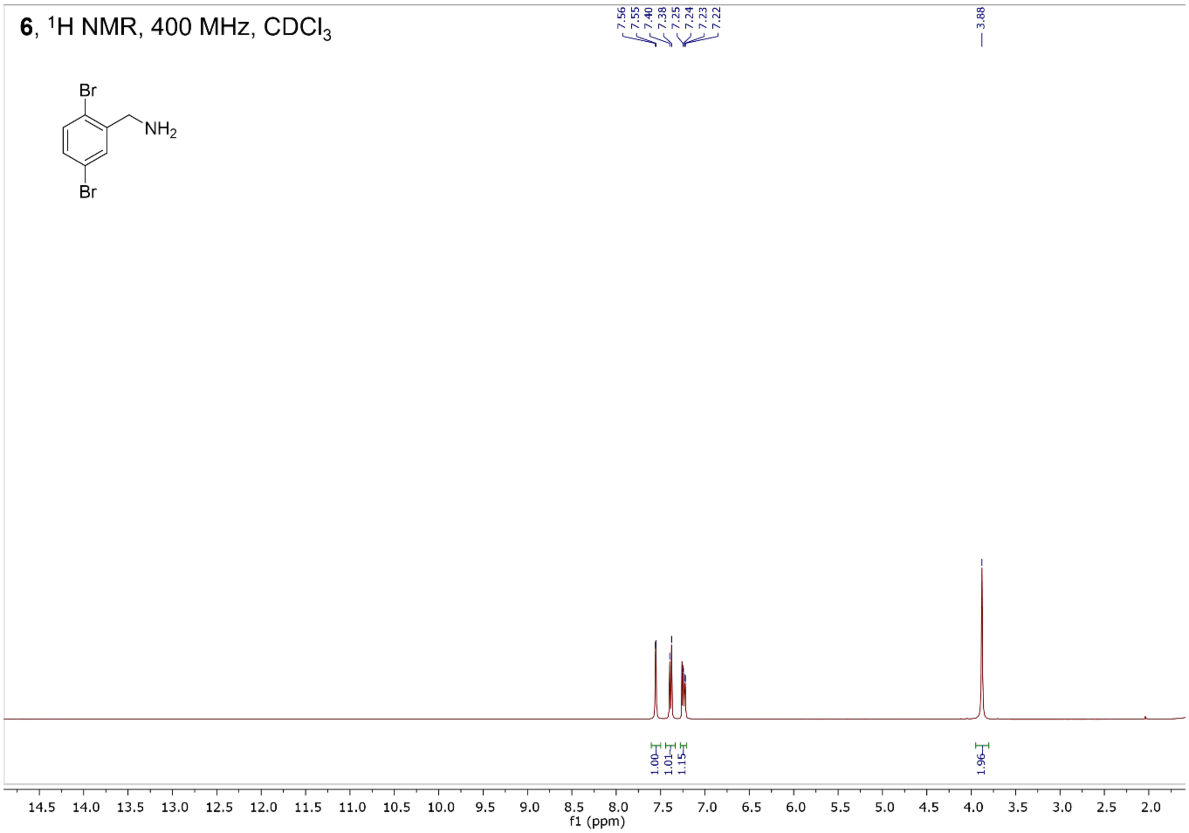
6, ^{1}H NMR, 400 MHz, $CDCl_3$
Br
NH2
Br
7.56
7.55
7.40
7.38
7.25
7.24
7.23
7.22
3.88
1.00
1.01
1.15
1.96
14.5 14.0 13.5 13.0 12.5 12.0 11.5 11.0 10.5 10.0 9.5 9.0 8.5 8.0 7.5 7.0 6.5 6.0 5.5 5.0 4.5 4.0 3.5 3.0 2.5 2.0
f1 (ppm)

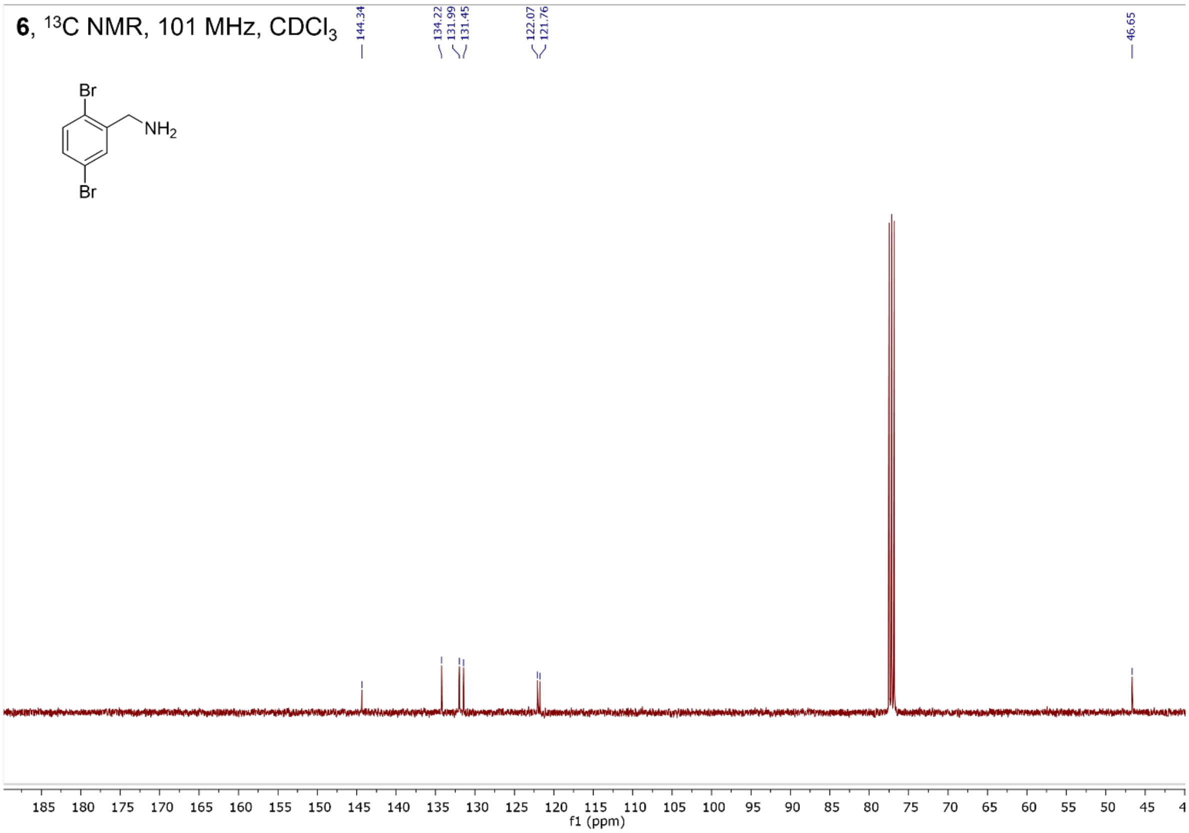
6, ^{13}C NMR, 101 MHz, $CDCl_3$
Br
NH2
Br
144.34
134.22
131.99
131.45
122.07
121.76
46.65
185 180 175 170 165 160 155 150 145 140 135 130 125 120 115 110 105 100 95 90 85 80 75 70 65 60 55 50 45 4
f1 (ppm)

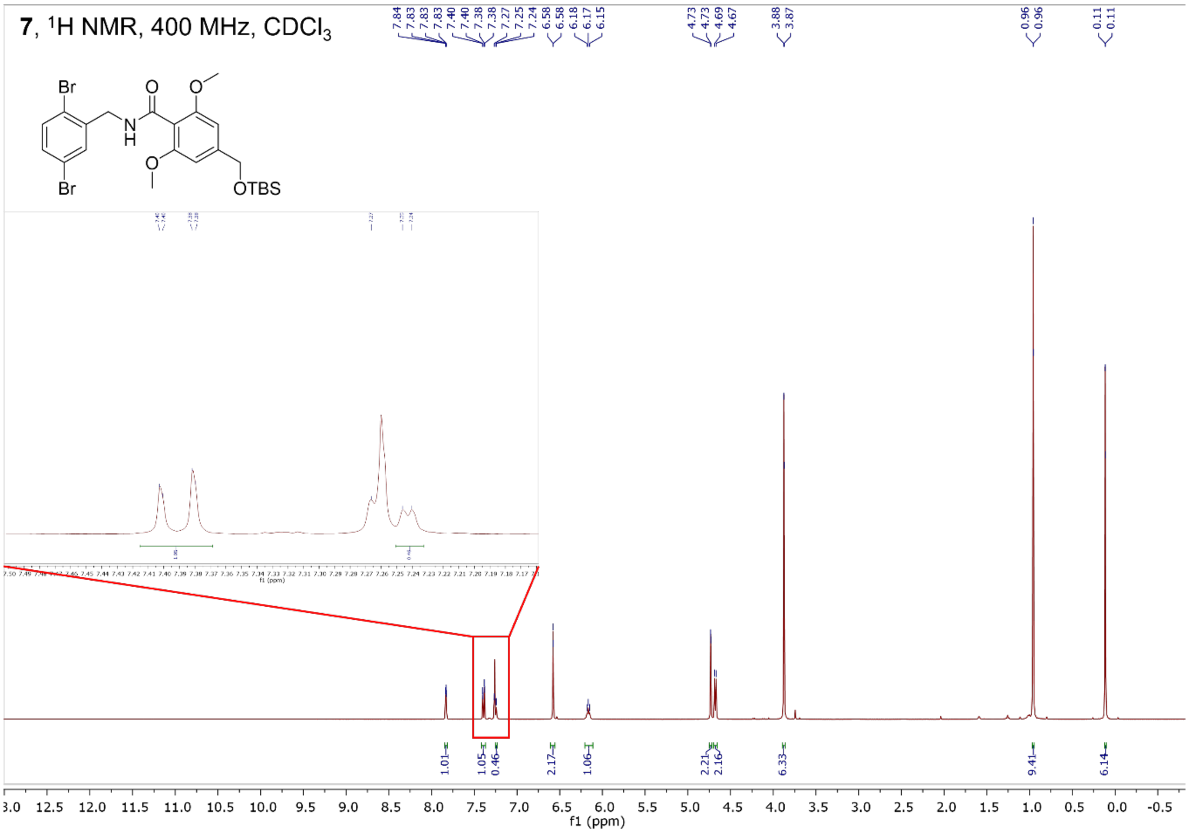
7, ¹H NMR, 400 MHz, CDCl₃
Br
O
O
N
H
O
Br
OTBS
7.84
7.83
7.83
7.83
7.40
7.40
7.38
7.38
7.27
7.25
7.24
6.58
6.58
6.18
6.17
6.15
4.73
4.73
4.69
4.67
3.88
3.87
0.96
0.96
0.11
0.11
1.01
1.05
0.46
2.17
1.06
2.21
2.16
6.33
9.41
6.14
f1 (ppm)

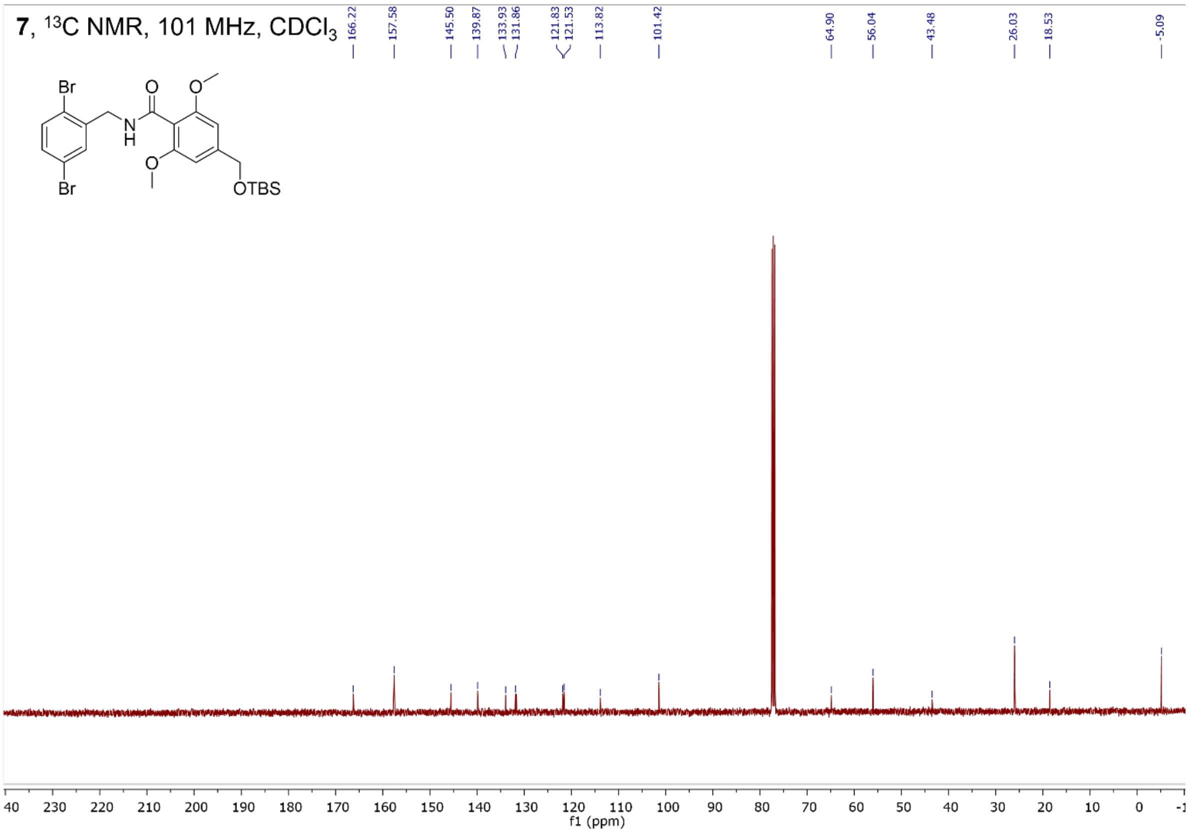
7, ¹³C NMR, 101 MHz, CDCl₃
166.22
157.58
145.50
139.87
133.93
131.86
121.83
121.53
113.82
101.42
64.90
56.04
43.48
26.03
18.53
-5.09
Br
O
O
N
H
O
Br
OTBS
f1 (ppm)

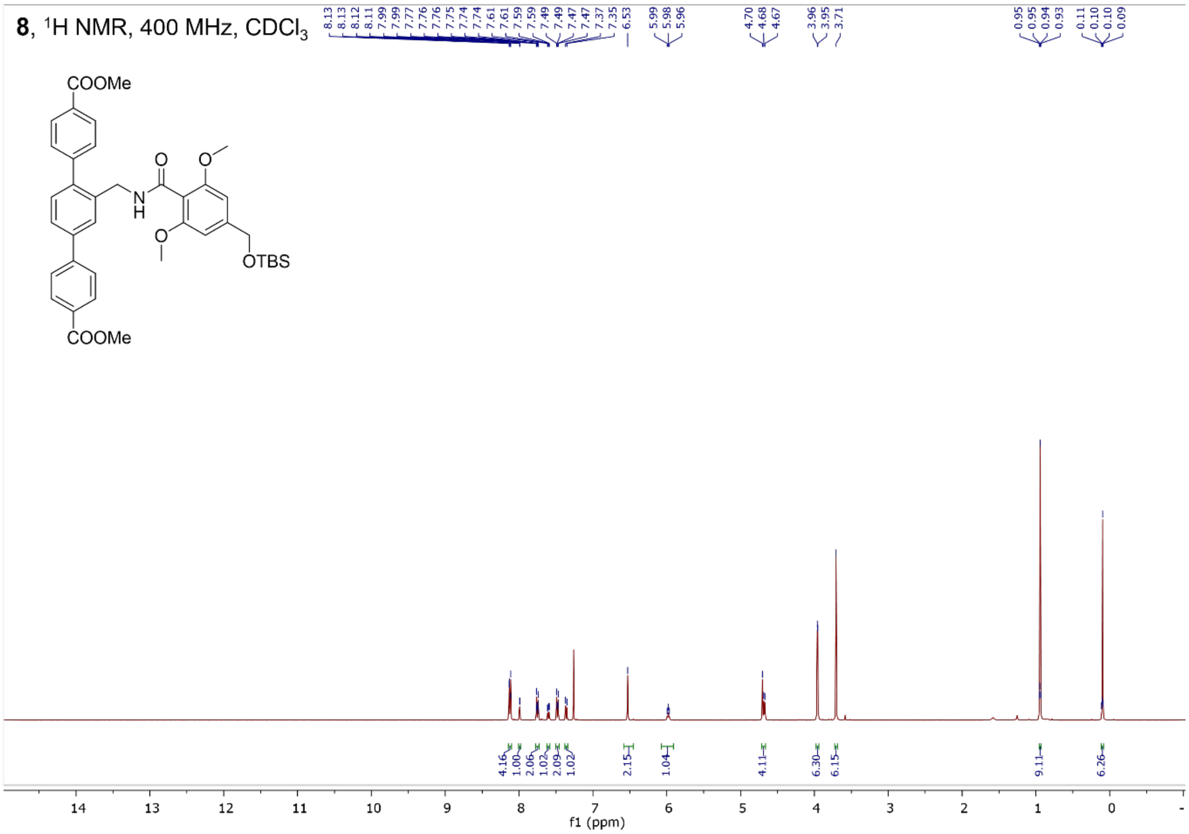
8, 1H NMR, 400 MHz, CDCl3
COOMe
O
O
N
H
O
OTBS
COOMe
f1 (ppm)

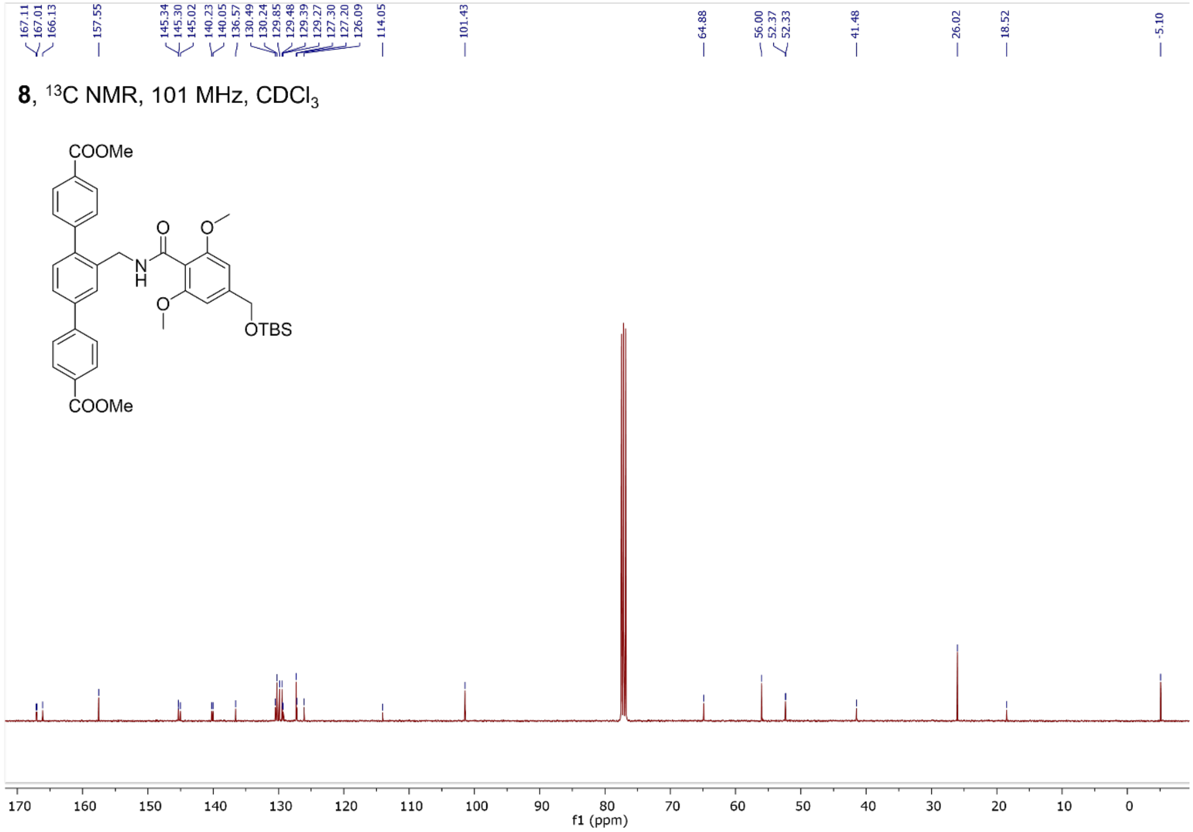
8, 13C NMR, 101 MHz, CDCl3
167.11
167.01
166.13
157.55
145.34
145.30
145.02
140.23
140.05
136.57
130.49
130.24
129.85
129.48
129.39
129.27
127.30
127.20
126.09
114.05
101.43
64.88
56.00
52.37
52.33
41.48
26.02
18.52
-5.10
COOMe
O
O
N
H
O
OTBS
COOMe
f1 (ppm)

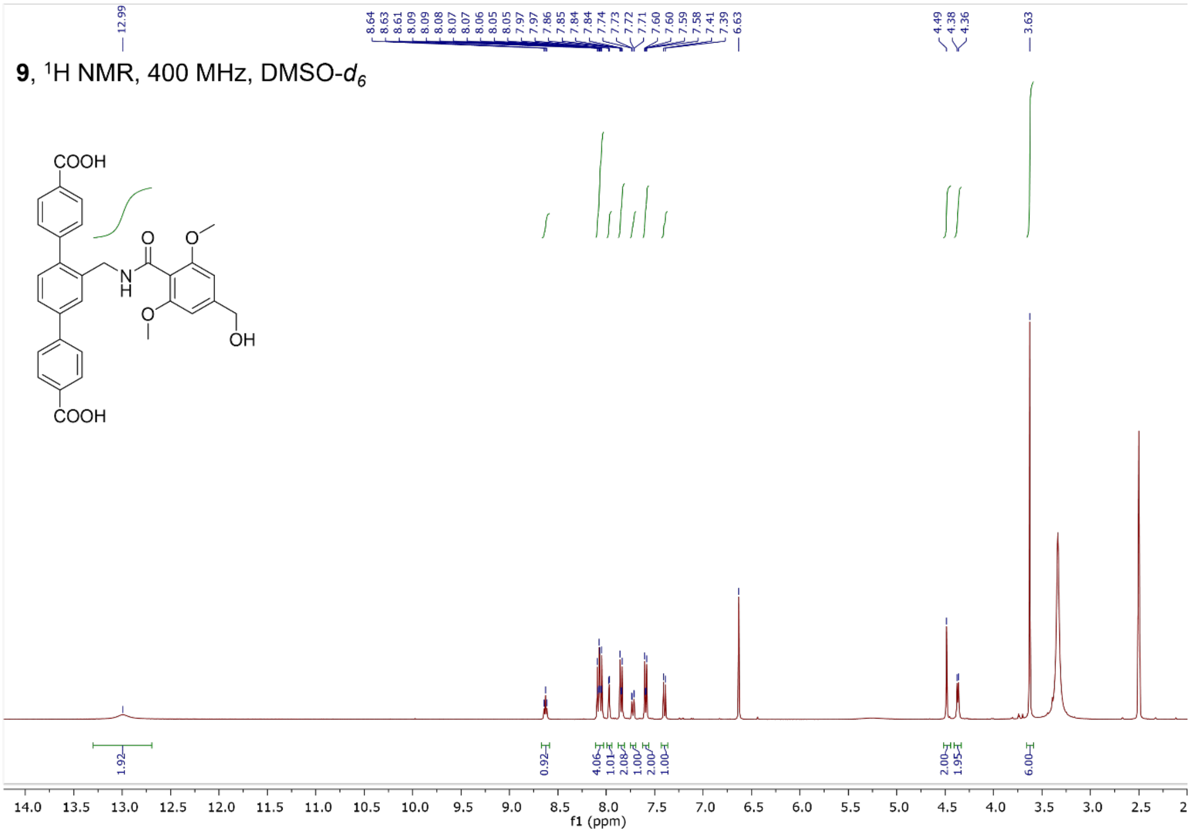
9, ¹H NMR, 400 MHz, DMSO-d6
COOH
COOH
OH
f1 (ppm)

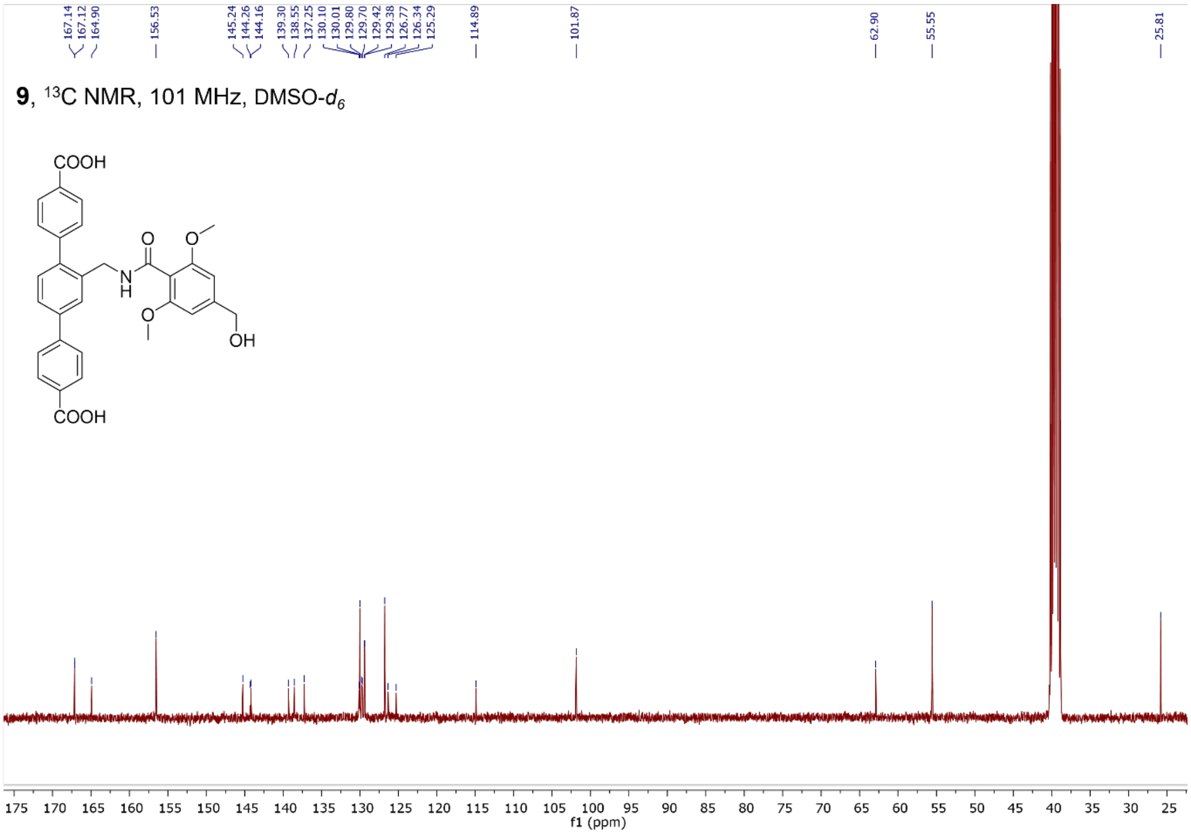
9, ¹³C NMR, 101 MHz, DMSO-d6
167.14
167.12
164.90
156.53
145.24
144.26
144.16
139.30
138.55
137.25
130.10
130.01
129.80
129.70
129.42
129.38
126.77
126.34
125.29
114.89
101.87
62.90
55.55
25.81
COOH
O
O
N
H
O
OH
COOH
175 170 165 160 155 150 145 140 135 130 125 120 115 110 105 100 95 90 85 80 75 70 65 60 55 50 45 40 35 30 25
f1 (ppm)

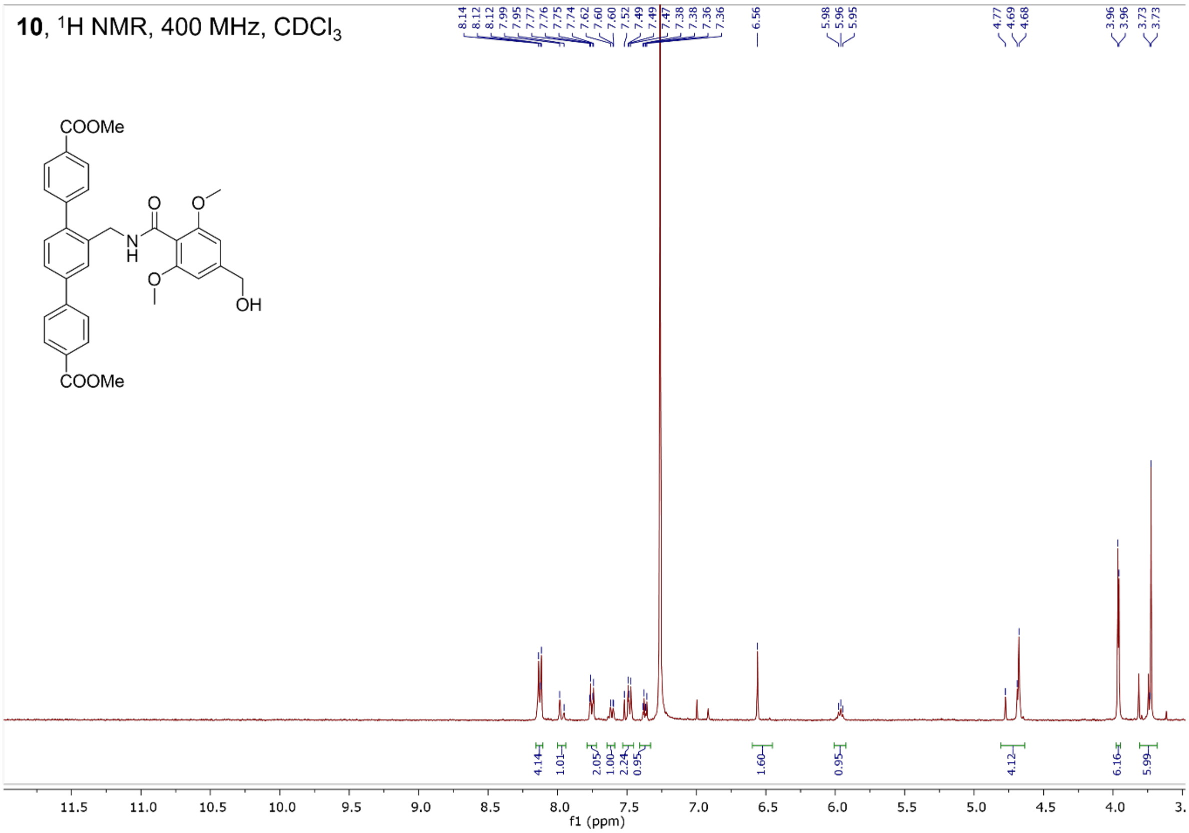
10, ¹H NMR, 400 MHz, CDCl3
8.14
8.12
8.12
7.99
7.95
7.77
7.76
7.75
7.74
7.62
7.60
7.52
7.49
7.49
7.47
7.38
7.38
7.36
7.36
6.56
5.98
5.96
5.95
4.77
4.69
4.68
3.96
3.96
3.73
3.73
COOMe
O
O
N
H
O
OH
COOMe
4.14
1.01
2.05
1.00
2.24
0.95
1.60
0.95
4.12
6.16
5.99
11.5 11.0 10.5 10.0 9.5 9.0 8.5 8.0 7.5 7.0 6.5 6.0 5.5 5.0 4.5 4.0 3.
f1 (ppm)

10, ^{1}H NMR, 400 MHz, DMSO-d_6

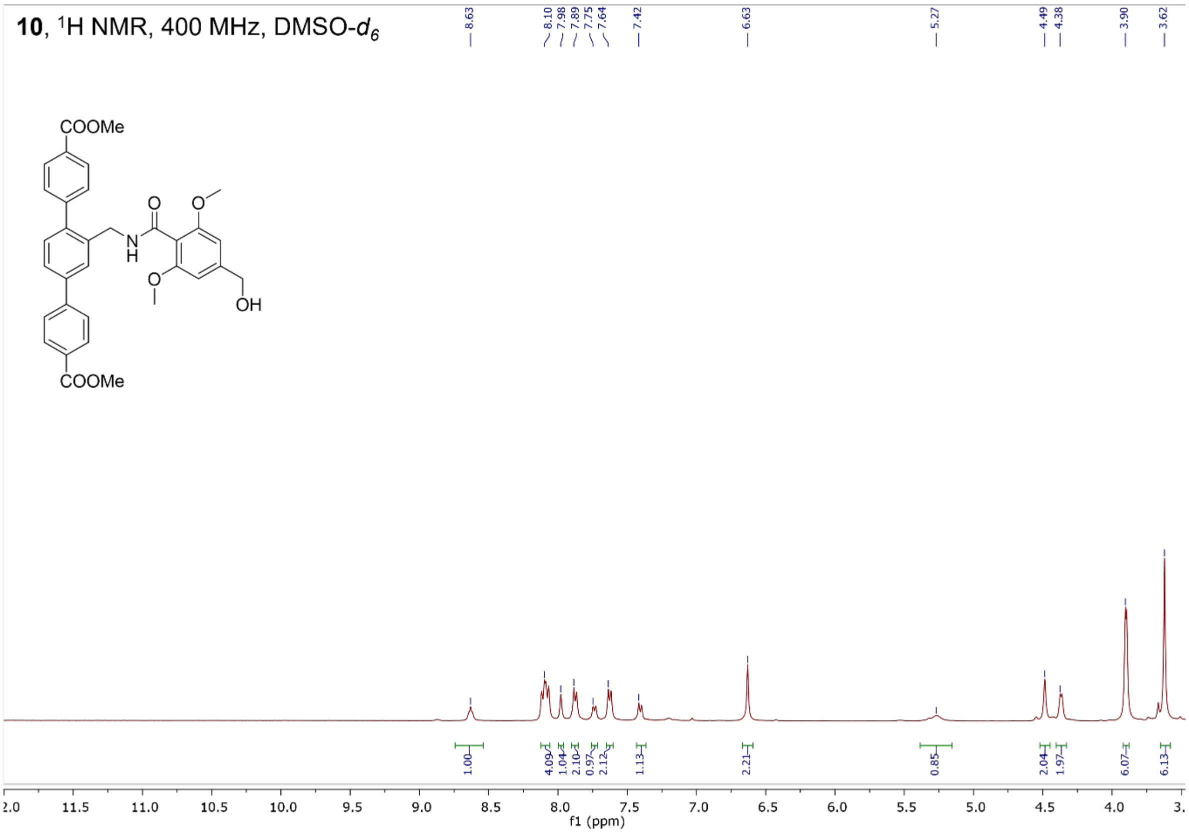

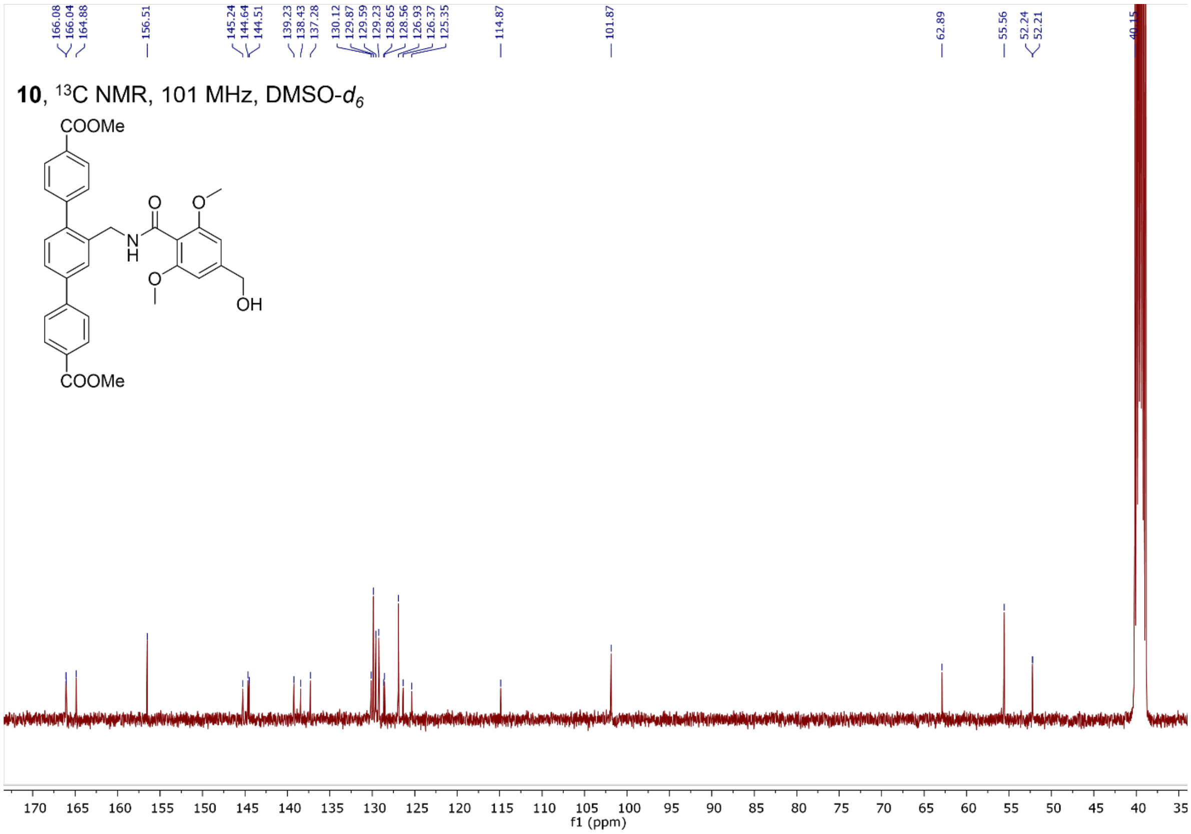
10, ¹³C NMR, 101 MHz, DMSO-d6
COOMe
COOMe
OH
f1 (ppm)

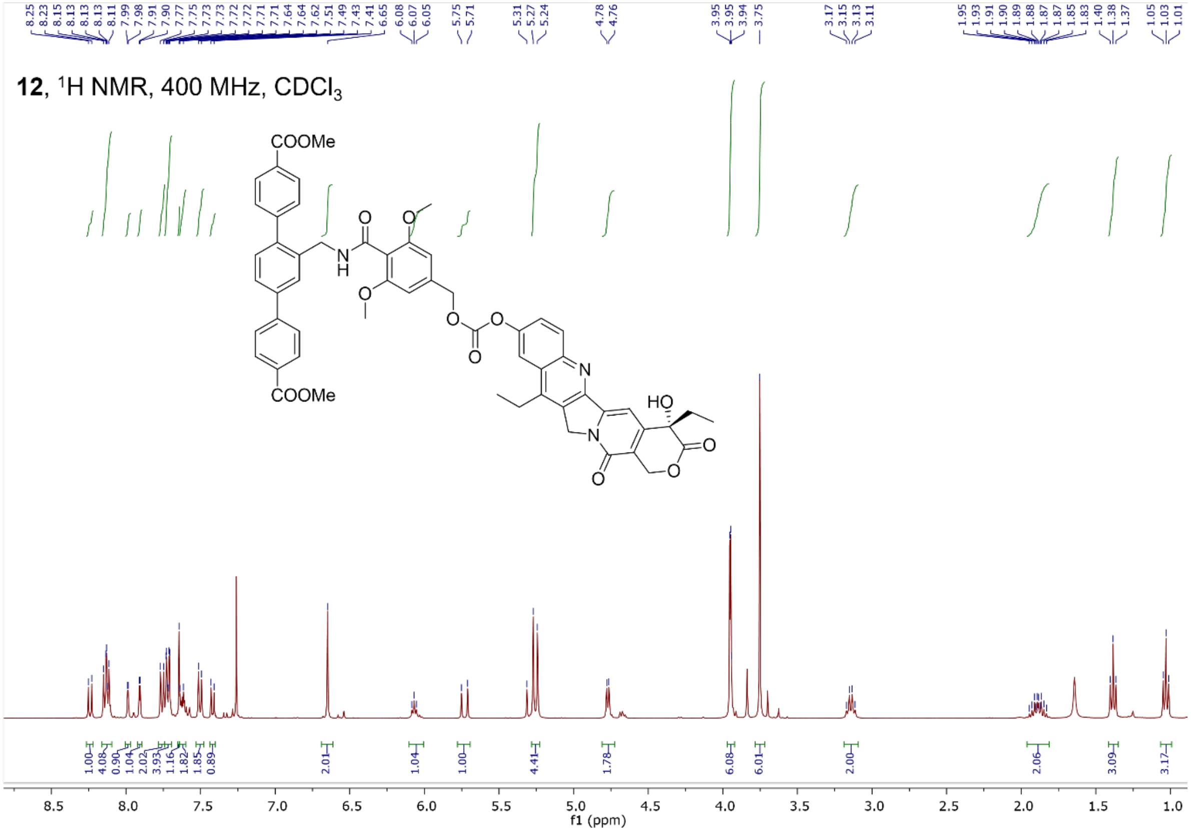
12, ¹H NMR, 400 MHz, CDCl3
COOMe
COOMe
HO
f1 (ppm)

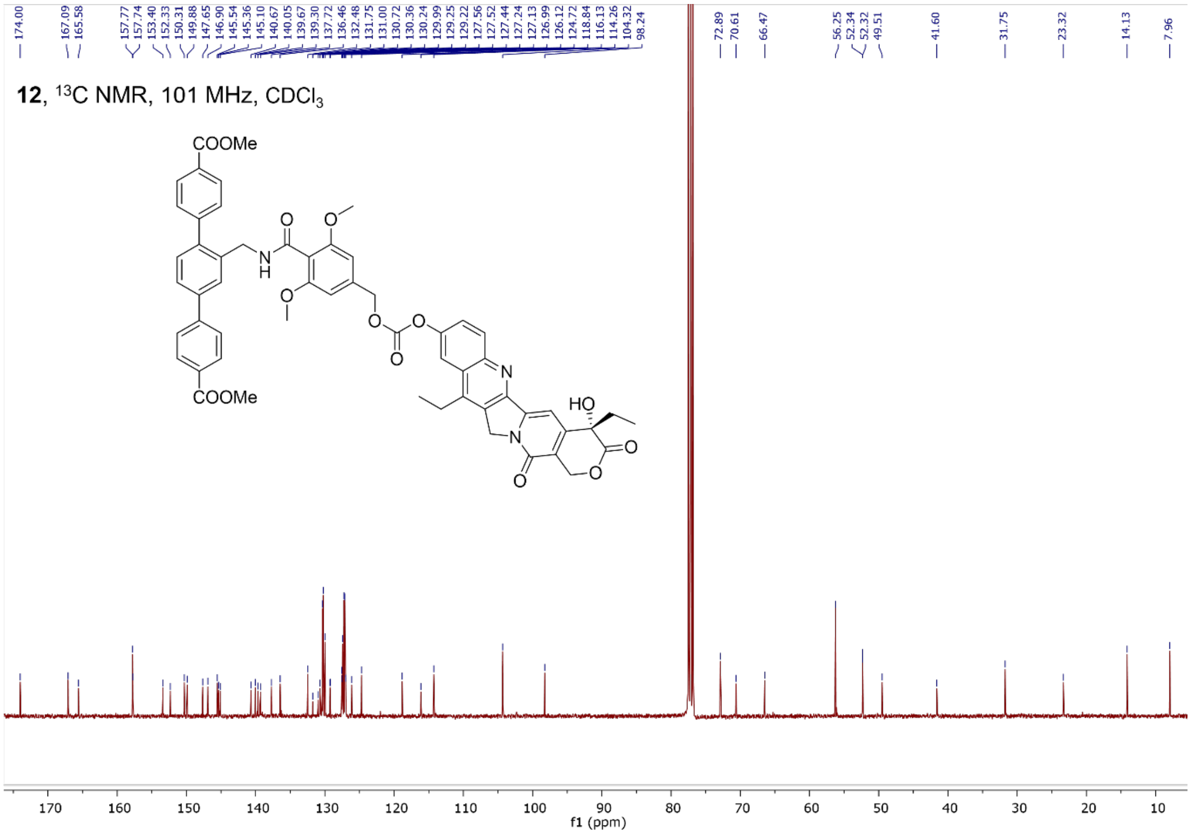
12, ^{13}C NMR, 101 MHz, $CDCl_3$
COOMe
COOMe
N
H
O
HO
N
N
174.00
167.09
165.58
157.77
157.74
153.40
152.33
150.31
149.88
147.65
146.90
145.54
145.36
145.10
140.67
140.05
139.67
139.30
137.72
136.46
132.48
131.75
131.00
130.72
130.36
130.24
129.99
129.25
129.22
127.56
127.52
127.44
127.24
127.13
126.99
126.12
124.72
118.84
116.13
114.26
104.32
98.24
72.89
70.61
66.47
56.25
52.34
52.32
49.51
41.60
31.75
23.32
14.13
7.96
f1 (ppm)

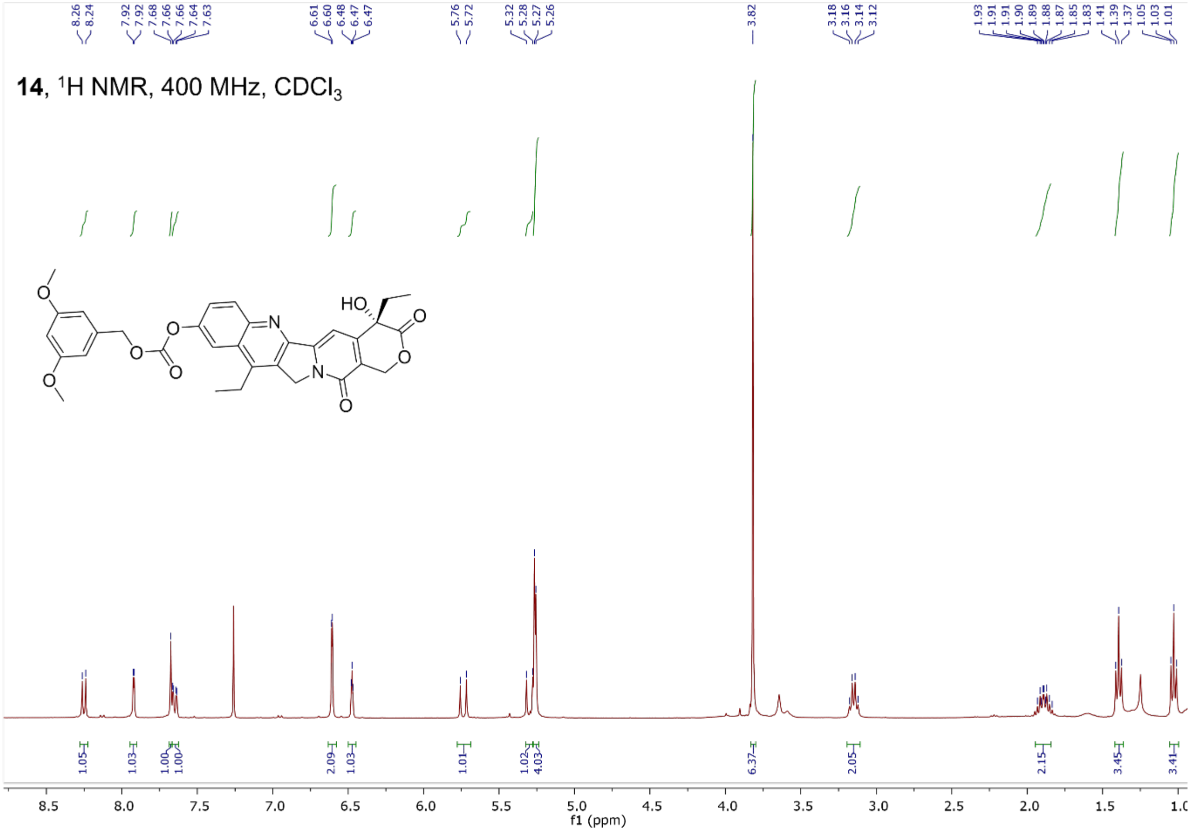
14, ^{1}H NMR, 400 MHz, $CDCl_3$
HO
N
N
O
8.26
8.24
7.92
7.92
7.68
7.66
7.66
7.64
7.63
6.61
6.60
6.48
6.47
6.47
5.76
5.72
5.32
5.28
5.27
5.26
3.82
3.18
3.16
3.14
3.12
1.93
1.91
1.91
1.90
1.89
1.88
1.87
1.85
1.83
1.41
1.39
1.37
1.05
1.03
1.01
1.05
1.03
1.00
1.00
2.09
1.03
1.01
1.02
4.03
6.37
2.05
2.15
3.45
3.41
f1 (ppm)

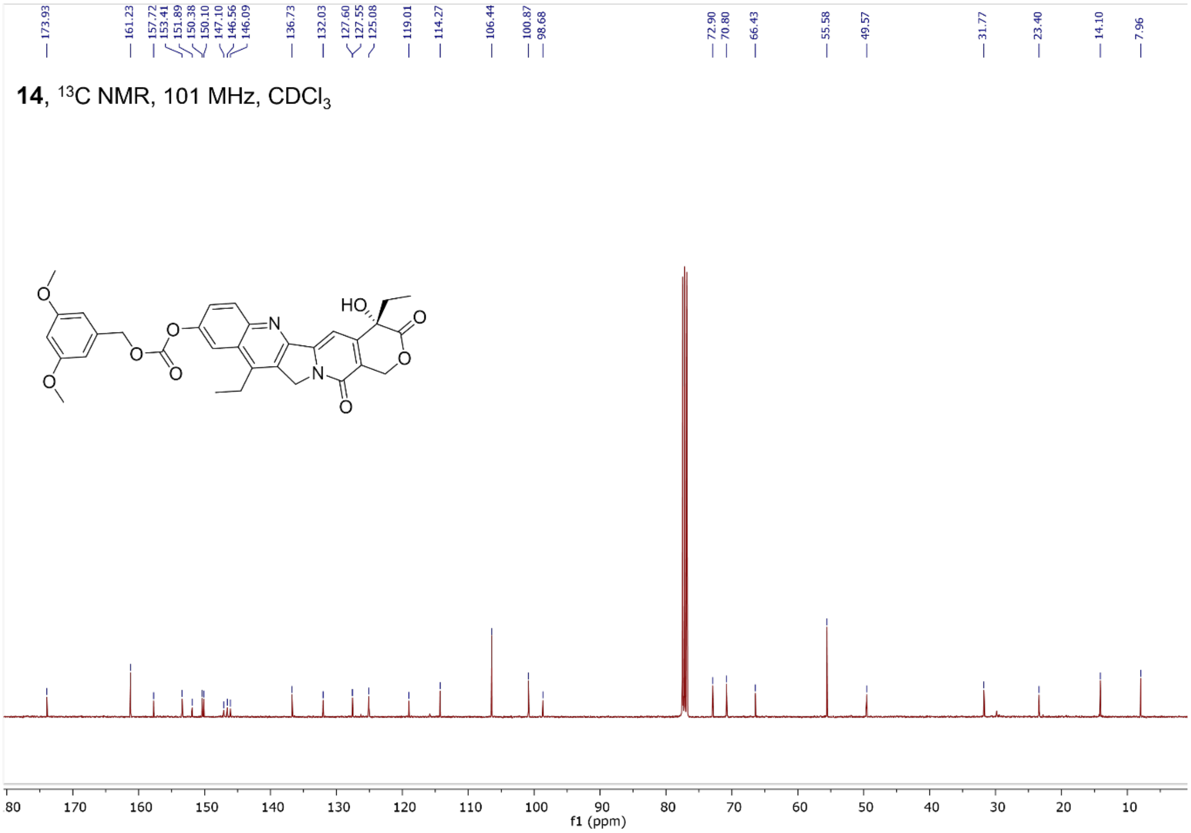

S8. References

(1) Fracaroli, A. M.; Furukawa, H.; Suzuki, M.; Dodd, M.; Okajima, S.; Gándara, F.; Reimer, J. A.; Yaghi, O. M., Metal–Organic Frameworks with Precisely Designed Interior for Carbon Dioxide Capture in the Presence of Water. *J. Am. Chem. Soc.* **2014,** *136* (25), 8863-8866.

(2) Ju, E.; Dong, K.; Chen, Z.; Liu, Z.; Liu, C.; Huang, Y.; Wang, Z.; Pu, F.; Ren, J.; Qu, X., Copper(II)–Graphitic Carbon Nitride Triggered Synergy: Improved ROS Generation and Reduced Glutathione Levels for Enhanced Photodynamic Therapy. *Angew. Chem. Int. Ed.* **2016,** *55* (38), 11467-11471.

(3) Ni, K.; Lan, G.; Chan, C.; Duan, X.; Guo, N.; Veroneau, S. S.; Weichselbaum, R. R.; Lin, W., Ultrathin Metal-Organic-Layer Mediated Radiotherapy-Radiodynamic Therapy. *Matter* **2019,** *1* (5), 1331-1353.

(4) Luo, T.; Nash, G. T.; Jiang, X.; Feng, X.; Mao, J.; Liu, J.; Juloori, A.; Pearson, A. T.; Lin, W., A 2D Nanoradiosensitizer Enhances Radiotherapy and Delivers STING Agonists to Potentiate Cancer Immunotherapy. *Adv. Mater.* **2022,** *34*, 2110588.
